# RedoxDB and RedEx: closing the DFT-experiment domain gap in redox potential prediction with transfer learning

**DOI:** 10.1039/d6sc05970h

**Published:** 2026-10-15

**Authors:** Dat M. Doan, Qilei Song, Anthony Kucernak, Kim E. Jelfs

**Affiliations:** a Department of Chemistry, Imperial College London, White City Campus London W12 0BZ UK k.jelfs@imperial.ac.uk; b Department of Chemical Engineering, Imperial College London, South Kensington Campus London SW7 2AZ UK

## Abstract

Organic redox-active molecules (ORMs) are promising components of aqueous electrolytes in redox flow batteries (RFBs), a scalable electrochemical energy storage technology designed to address the intermittency of renewable energy. However, the development of aqueous ORMs is challenged by the need to satisfy multiple property requirements, including redox potential (*E*°), solubility and stability under operating conditions. Among these, redox potential is a critical parameter, as the thermodynamic stability window of water under standard conditions is limited to 0.000–1.229 V, making accurate prediction essential to designing new ORMs. Conventional computational approaches for estimating *E*°, such as density functional theory (DFT), can exhibit errors in the range of 0.2–0.5 V relative to experiment. Here, we present RedoxDB, a new database of >7500 DFT-calculated redox potentials for aqueous ORMs, which we use to pre-train a prediction model. Our prediction model, RedEx, is an ensemble graph attention network (GAT) employing transfer learning for the accurate prediction of experimental redox potential values, to close the domain gap between DFT and experiment. The model is subsequently fine-tuned with a curated dataset of experimental redox potential values taken from the literature. We demonstrate that this transfer learning strategy improves predictive performance, achieving a mean absolute error of 0.057 V, significantly reducing the domain gap between DFT and experiment. The attention mechanism of the GAT model also provides interpretability, connecting predictions directly to chemically important structural motifs. We envisage this model will help facilitate the design of novel redox couples for aqueous organic RFBs. The associated code is available at https://github.com/datmdoan/RedEx.

## Abstract 2

RedoxDB is a new database of DFT-calculated redox potentials for aqueous organic redox molecules. RedEx combines graph neural networks with transfer learning from RedoxDB to predict experimental potentials with a mean absolute error of 0.057 V.

## Graphical abstract

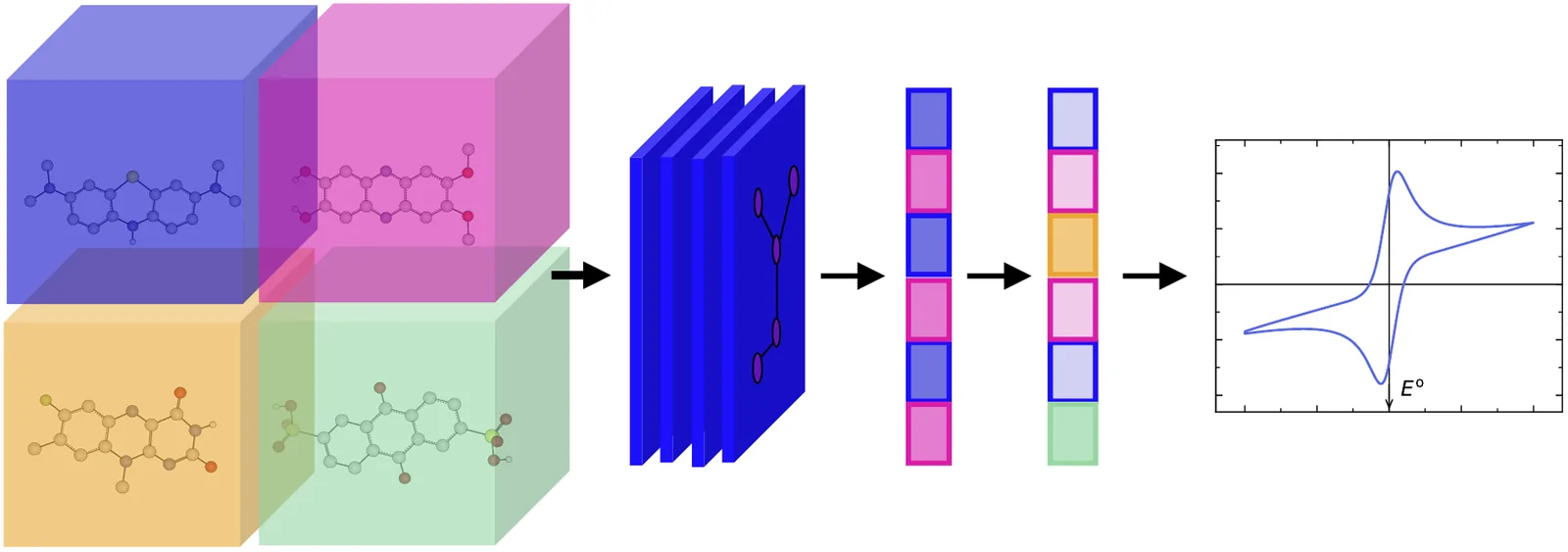

## 1 Introduction

With an increasing demand for renewable energy storage solutions, redox flow batteries (RFBs) have been used to meet intermittent energy demands, particularly for grid-scale applications.^1,2^ RFBs pump electrolytic solvents containing redox-active species to generate energy in charge–discharge cycles and a major advantage lies in the decoupling of power and energy capacity, allowing independent scaling of tank size and cell stack. Non-aqueous RFBs (NARFBs) use organic solvents that offer high theoretical voltages, but are plagued by operational challenges, from safety to substantial resistive losses and typically the requirement to keep the system oxygen and water free.^3,4^ In contrast, aqueous redox flow batteries (ARFBs) use water as the primary solvent, offering an intrinsically safer and lower cost approach.^5^ Despite the commercial maturity of all-vanadium RFBs, the growing vanadium cost and inconsistent, less sustainable supply have created a need for next-generation RFBs.^6^ Consequently, aqueous organic RFBs (AORFBs), composed of earth-abundant elements, address these challenges by offering a potentially more sustainable, cost-effective and higher power solution.

A major challenge for AORFB development is the molecular engineering of the organic redox-active molecules (ORMs) for the aqueous electrolytes. Many ORMs have been derived biomimetically^7,8^ and synthesised with prominent classes being quinones,^9^ phenazines,^10^ viologens,^11^ alloxazines^12^ and nitroxide radicals.^13^ The difficulty of designing appropriate ORMs for RFBs lies in multi-faceted design requirements, making for a challenging multi-variable optimisation problem. Key parameters include the redox potential, solubility, operating pH, redox kinetics, stability, cost and ease of synthesis.^14^

The redox potential (*E*°) is a critical property of a RFB, directly influencing the maximum storage capacity. The cell voltage is directly linked to the potential difference between the redox potentials associated with the catholyte and anolyte. A challenge and limitation of AORFBs is that water sets a thermodynamic stability window of 0.000–1.229 V. However, the slow kinetics of water reduction and oxidation, and judicious choices of electrode materials on which those reactions are slow, can extend the upper point of the window up to 1.6 V.^14^ Different molecular classes also undergo distinct redox reactions, including multi-step and multi-electron processes. To design novel AORFB electrolytes, the redox potential of an ORM must be predicted accurately.

The tunable nature of organic molecules has enabled experimental researchers to explore ORM chemical space *via* a ‘trial-and-error’ approach, usually by the introduction of electron-withdrawing groups (EWGs) or electron-donating groups (EDGs) to tune specific properties in a series of iterative computational simulations, followed by attempted synthesis and characterisation.^9,15^ However, such an approach is time-consuming and often reveals a trade-off against other properties such as solubility or chemical stability.^16,17^

Computational methods, particularly density functional theory (DFT) calculations have become a powerful tool for materials design, with numerous studies using high-throughput virtual screening to navigate the vast chemical space. Theoretically, DFT calculations can be used to predict *E*° values by employing a Born–Haber thermodynamic cycle to calculate the Gibbs free energy of reaction in solution 
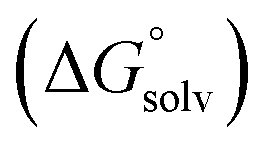
 and then using the Nernst equation, typically resulting in mean absolute errors (MAEs) of 0.2–0.5 V for lower cost methods.^18–20^ Other computational studies employ proxy metrics for modelling *E*°, for example, Er *et al.* developed a DFT-level *E*° calibration model to explore the positional effects of EDGs and EWGs for a library of over 1700 quinone-type derivatives.^21^ However, DFT calculations are computationally expensive, making the exhaustive exploration of chemical space infeasible, although with reasonable computing resource, large computational databases can still be constructed.

Recent efforts in the context of AORFBs have led to the curation of two notable repositories, including SOMAS, an open-source solubility database for ORMs^22^ and RedDB, a large computational library comprising 15 000 ORM redox couples.^23^ In the development of RedDB, Sorkun *et al.*^23^ employed high-throughput DFT calculations to model the proton-coupled electron transfer (PCET) reactions of 52 core quinone and aza-aromatics scaffolds, exhaustively enumerated with 5 R-groups, –SO_3_H, –COOH, –NH_2_, –OH and –F. While providing a large dataset, these values represent DFT-calculated reaction energies, a theoretical proxy for *E*° and focus on a limited number of substituents.

The emergence of machine learning (ML) has enabled researchers to use large datasets to vastly accelerate the discovery of novel molecules in fields such as drug discovery and for property prediction with DFT-level accuracy, at a fraction of the cost.^24,25^ Graph neural networks (GNNs) have proven particularly adept at molecular property prediction in chemistry and materials science. By representing molecules as graphs, where atoms are nodes and bonds are edges, GNNs can learn the complex relationship between molecular topology and intrinsic properties directly from the structure.^26,27^

Building upon RedDB, Sorkun *et al.* introduced RedPred, an ensemble ML model incorporating artificial neural networks, random forest and graph convolution networks (GCNs) to predict redox reaction energies.^28^ RedPred achieves strong DFT-level predictions of 0.1 eV MAE but its predictive quality is inhibited by the underlying limited structural diversity in RedDB. Consequently, the model suffers from significant relative error percentages up to 90% when tasked with out-of-distribution (OOD) molecules. Parallel to this, Chaka *et al.* developed MolGAT, an augmented graph attention network (GAT) trained on RedDB, inheriting the same structural biases and continuing to rely on theoretical reaction energies as a proxy for *E*°.^29^ Fedorov *et al.* presented a multi-solvent GNN model to predict DFT-calculated redox potentials, achieving an MAE of 0.2 eV.^30^ Their results highlighted the challenge of accurately modelling even computational values, let alone experimental measurements, usually measured in a wide range of conditions, which can affect the reversibility. Predicting the *E*° of OOD molecules is also inherently challenging due to limited training coverage of chemical space and potential variations in the underlying redox mechanism. Furthermore, in the laboratory, electrochemical potentials are measured in a wide range of conditions, which affect the reversible electrochemical potential through the Nernst equation. The measurement conditions need to be deconvoluted to obtain the potential under standard conditions, which is not trivial, especially if the redox molecule contains acid or base functionality.

Here, we introduce RedoxDB, a new, structurally diverse database of more than 7500 DFT-calculated *E*° values, developed to support ML for redox potential prediction. It spans 57 molecular scaffolds, including quinones, phenazines, alloxazines, and thiazine derivatives, the latter being a promising but relatively underexplored class of ORMs with comparatively high redox potentials.^31–34^ RedoxDB was generated using an automated high-throughput DFT workflow applied to a virtually generated library of ORMs. To connect this computational space to experiment, we also curated a literature dataset of 264 experimental *E*° values, measured by cyclic voltammetry (CV). Both datasets can be found at https://github.com/datmdoan/RedEx/tree/main/data.

Built on these datasets, we introduce RedEx, an ensemble GAT model that uses transfer learning to predict experimental *E*° values (Fig. 1). Transfer learning leverages abundant low-fidelity computational data to improve prediction in a data-sparse high-fidelity experimental domain,^35,36^ and has proved effective for molecular property prediction with GNNs.^37,38^ Consistent with this, we found fine-tuning on our curated experimental data reduced the domain gap and enabled RedEx to outperform models trained without pre-training. We further incorporate uncertainty quantification and interpretability analysis, providing a robust framework for the rapid design of next-generation aqueous ORMs.

**Fig. 1:**
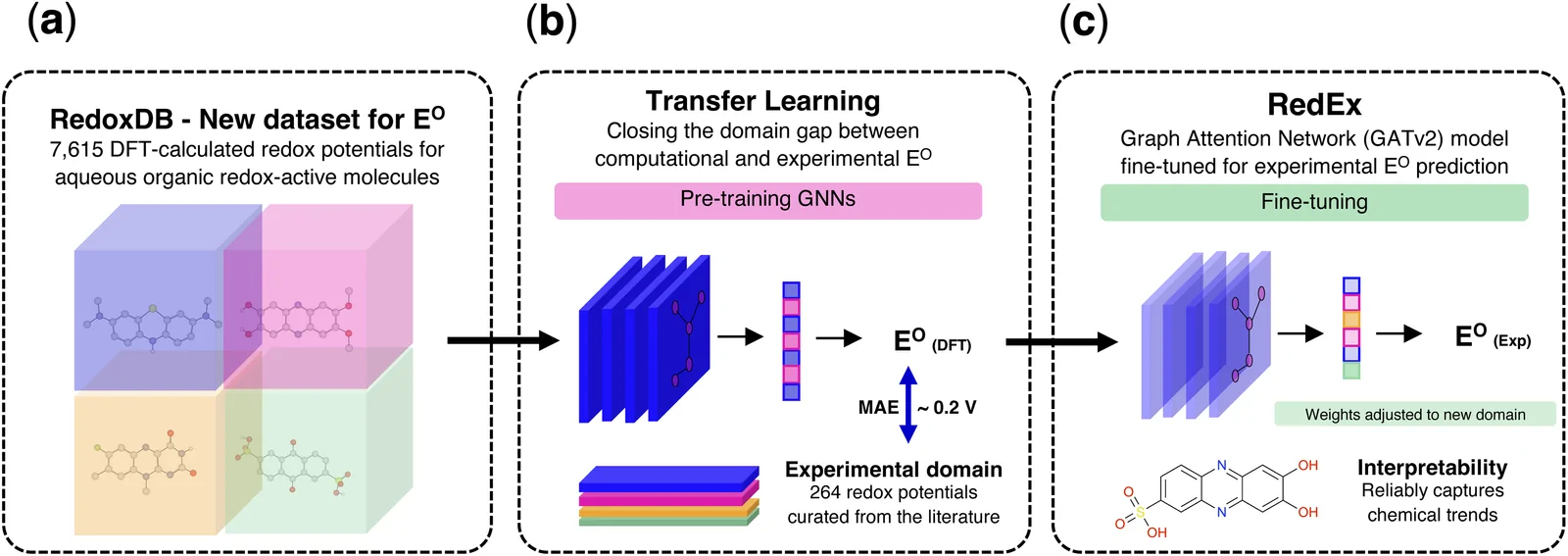
Overview of the RedEx workflow. (a) RedoxDB, a chemically diverse dataset containing 7615 DFT-calculated *E*° values and associated electronic properties for aqueous ORMs. (b) Transfer learning is used to bridge the domain gap between DFT and experiment. GNN models are firstly pre-trained on RedoxDB and then fine-tuned using a curated set of experimental measurements from the literature. (c) RedEx, an ensemble GAT model fine-tuned on experimental data, enabling accurate prediction of experimental *E*° values, while providing interpretable insights into chemical structure.

## 2 Methods

### 2.1 RedoxDB

#### 2.1.1 Virtual combinatorial library

Robust generalisation in ML models requires training on chemically diverse datasets, which remain limited for aqueous ORMs, both in functionalisations and scaffold types. To overcome this limitation, we constructed a new virtual combinatorial library of aqueous ORMs spanning quinones, phenazines, alloxazines and thiazines, encompassing one-, two- and three-ring as well as extended conjugated systems. Such structural features are known to dictate redox potentials and underpin stability and reversibility, as widely reported in the literature.^14^ A total of 57 core scaffolds were designed based on reported redox-active molecules, each representing a distinct structure within each heterocyclic class. The full list of scaffold and functional groups used in this study is provided in Section S1.1 and S1.2. An RDKit^39^-based tool was developed to modify the SMILES of each scaffold with 27 functional groups (FGs). Each FG was attached at every chemically valid position of the scaffold, generating singly substituted derivatives. The tool checks implicit valence, removes duplicates *via* canonicalisation and prevents structural rearrangements, ensuring chemical validity of the generated SMILEs. The selected set of 27 functional groups spans common EWGs and EDGs, as well as FGs previously reported in the literature.^40–46^ The resulting virtual library captures a diverse region of chemical space, relevant to existing ORM motifs. The custom enumeration tool is available at https://github.com/datmdoan/compchem-tools.

#### 2.1.2 Redox potential calculation

The redox potential of an ORM is thermodynamically related to the change in the reaction free energy 
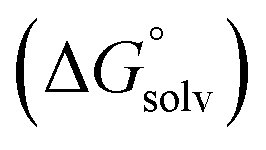
 of the corresponding redox reaction. Here, we consider the experimentally relevant reduction mechanisms reported in the literature. We model the two-electron, two-proton PCET reactions for quinones,^47^ phenazines^48^ and alloxazines^49^ and single-electron transfer (ET) reduction for thiazine structures.^50,51^ Each molecule is assigned a single predefined redox transformation based on its scaffold class and alternative redox processes are not considered. These reactions are modelled using separate Born–Haber cycles, as shown in Fig. 2a. The reaction free energy for the PCET is be calculated as:1
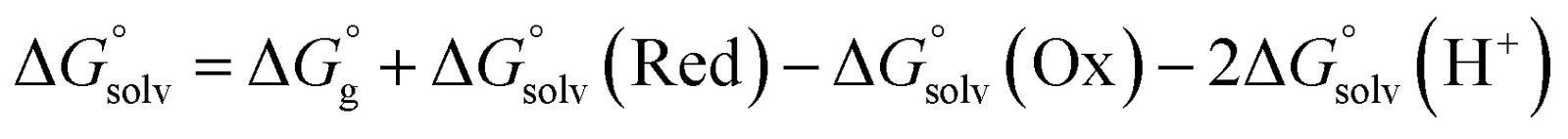
where 
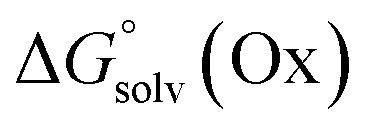
 and 
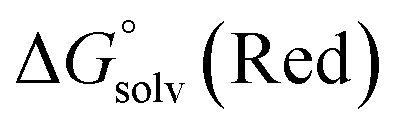
 are the solvation free energies of the oxidised and reduced molecular forms respectively. 
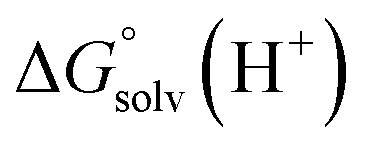
 is the solvation free energy of the proton in water, which has a value of −1105 kJ mol^−1^.^52^ The free energy of the electron is assumed to be zero.^52^ For the ET reactions, no proton is involved and thus the proton solvation free energy is not included. The corresponding reaction free energy for ET reactions are calculated as:2
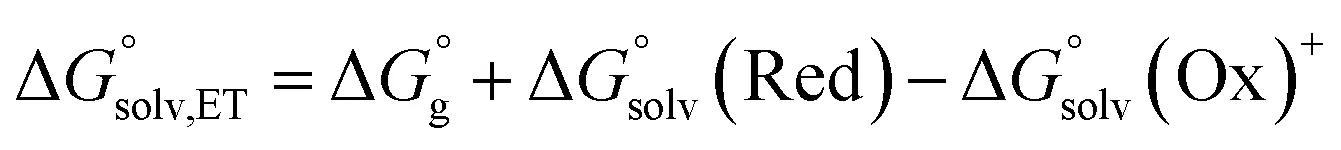


**Fig. 2:**
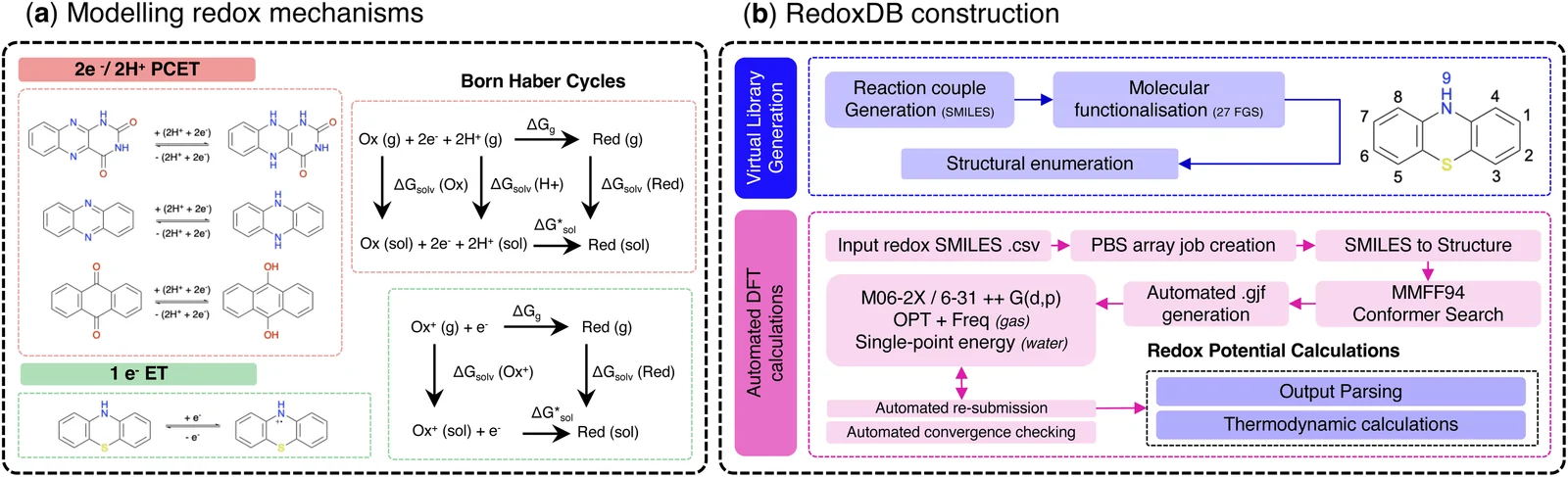
Overview of the construction of RedoxDB, a chemically diverse dataset of 7615 DFT-calculated *E*° values and associated electronic properties for aqueous ORMs. (a) Redox reactions and corresponding Born–Haber thermodynamic cycles used for the two-electron/two-proton PCET and one-electron ET reaction classes. (b) RedoxDB construction methodology, covering the virtual library generation and automated DFT calculations.

The resulting reaction energy changes were then converted to a reduction potential relative to the standard hydrogen electrode (SHE) according to:3
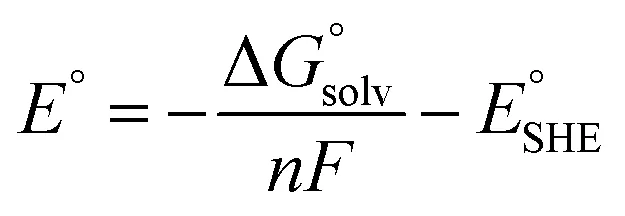
where *n* is the number of electrons transferred, *F* is the Faraday constant and 
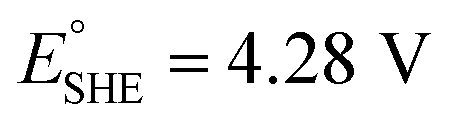
 is the absolute potential of the SHE.^52^ All computed redox potentials are therefore reported in volts *versus* SHE.

#### 2.1.3 Density functional theory calculations

To model PCET reactions with DFT, molecular structures corresponding to both the oxidised and reduced forms of each redox couple are required. For quinone, phenazine and alloxazine SMILES generated in the combinatorial library, reduced SMILES representations were produced from their oxidised counterparts using a custom RDKit-tool which can be found at https://github.com/datmdoan/compchem-tools. For the ET reactions of thiazines, the workflow automatically handles radical generation from SMILES.

All DFT calculations were performed using the Gaussian16 software package.^53^ These calculations were run on the Imperial College CX3 high-performance cluster (HPC) using a custom Python automated DFT workflow, accessible at https://github.com/datmdoan/compchem-tools. The workflow generates job-array scripts from an input CSV containing redox couple SMILES and manages all stages of the computational pipeline, including file generation, job submission, error handling and automated re-submission of failed calculations, as shown in Fig. 2b.

For each redox couple, the canonicalised oxidised and reduced SMILEs strings were converted to 3D geometries using RDKit. Ten conformers were generated and subsequently optimised using the MMFF94 force field. The lowest energy conformer was retained for DFT calculation, and Gaussian16 input files were generated using Pymatgen.^54^ Geometry optimisation and harmonic vibrational frequency calculations were carried out at the M06-2X/6-31++G(d,p) level of theory, which has been shown to provide good agreement with experimental *E*° values for ORMs.^19,55,56^ To further validate this choice, we benchmarked M06-2X/6-31++G(d,p) against B3LYP-D3/6-31++G(d,p) using curated experimental data. M06-2X showed improved agreement with experiment, particularly for thiazine single electron transfer reactions (see Section S1.3). Following geometry optimisation, harmonic frequency calculations were used to confirm *via* automated convergence checks, that all stationary points corresponded to local minima and contained no imaginary frequencies. Any failed calculations were re-submitted automatically and molecules that did not converge after repeated submissions (∼6%) were excluded from RedoxDB. Solvation effects were incorporated through single-point energy (SPE) calculations on the optimised geometries using the solvation model based on the density (SMD) implicit solvation model with water as the solvent. Thermal corrections to the Gibbs free energy obtained from the frequency calculations were applied to the single-point energies to obtain aqueous-phase free energies for both the oxidised and reduced species. The resulting *E*° values were subsequently calculated from the parsed outputs according to the thermodynamic cycle described in Fig. 2. This workflow yielded a computational dataset of 7615 DFT-calculated *E*° values, with associated electronic and thermodynamic properties.

### 2.2 Experimental data

The experimental *E*° dataset was complied manually from literature reports of aqueous *E*° values for ORMs, with a significant portion sourced from Gao *et al.*^57^ In total, the dataset comprises 264 cyclic voltammetry measurements for quinone (*n* = 202), phenazines (*n* = 35), alloxazines (*n* = 20) and thiazines (*n* = 7) and their distributions can be found in Fig. 3a. Thiazine dyes, although experimentally well established, were excluded from both datasets because their redox chemistry could not be modelled consistently (see Section S1.4). The t-SNE (t-distributed stochastic neighbour embedding) projection of the experimental dataset onto RedoxDB is shown in Fig. 3b.

**Fig. 3:**
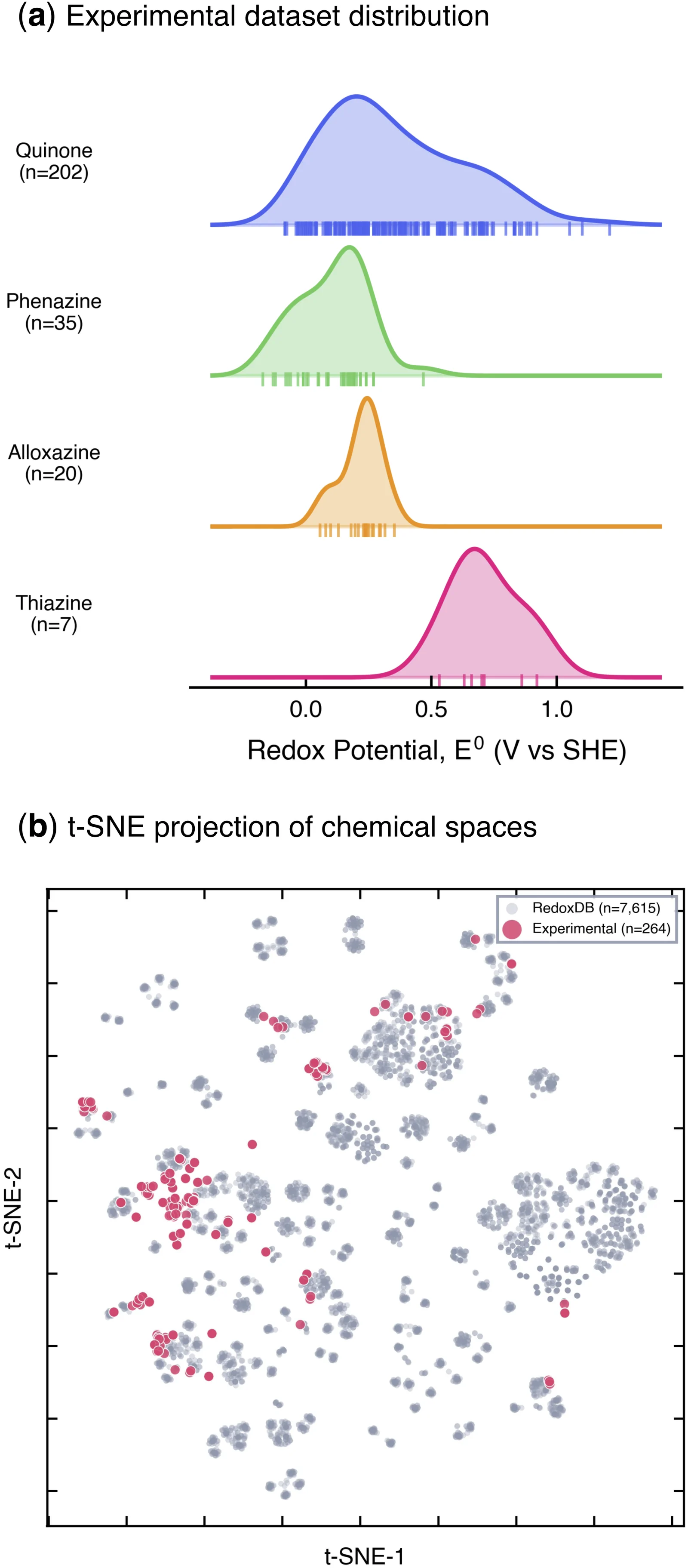
(a) Distribution of experimental redox potentials for the four molecular classes (*n* = 264). Each row shows a kernel density estimation (KDE) for one class, with tick marks denoting individual measurements. (b) t-SNE embedding of Morgan fingerprints for the RedoxDB dataset (*n* = 7615), coloured by molecular class. The t-SNE highlights the structural diversity and distinct clusters of the quinones, phenazines, alloxazines and thiazines.

In several cases, multiple measurements were available for the same molecule, arising from different literature sources or solvent conditions, with noticeable discrepancies between some reported values. To enable direct comparison with DFT-calculated redox potentials, all experimental values were adjusted *versus* the SHE and pH values were adjusted to 0. *E*° values not already reported *versus* the SHE, were converted from their original reference electrode to the SHE using:

4 *E*_*vs.* SHE_ = *E*_*vs.* ref_ + *E*_ref → SHE_,

where *E*_*vs.* ref_ is the reported potential *versus* the original reference electrode and *E*_ref → SHE_ is the appropriate reference-electrode conversion constant. For PCET redox reactions, potentials measured at non-zero pH were further adjusted to pH 0 according to the Nernst relation at 298 K:5
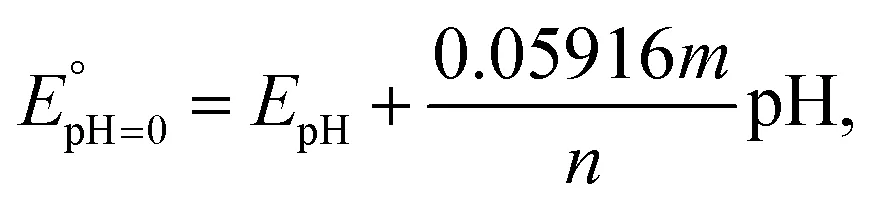
where *m* is the number of protons transferred, *n* is the number of electrons transferred, and *E*_pH_ is the experimentally reported potential at the measured pH.^52^ For the PCET reactions for quinones, phenazines, and alloxazines, this reduces to:6
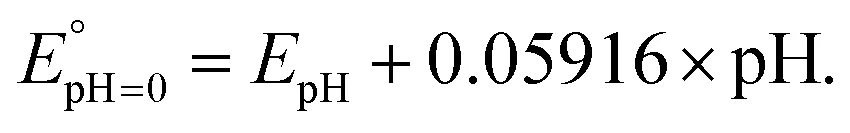


All experimental potentials used in this work are therefore reported in V *versus* SHE at pH 0, under the assumption that neither the oxidised nor reduced forms of the redox couple have a pKa between the measured pH and pH = 0.

### 2.3 Development of the RedEx model for experimental redox potential prediction

#### 2.3.1 Pre-training

Each molecule in RedoxDB was represented as a molecular graph, generated from its reactant SMILES using RDKit,^39^ with atoms corresponding to nodes and bonds to edges. Product structures were not provided as model inputs. We tested 5 GNN architectures; graph convolutional network (GCN),^58^ graph isomorphism network (GIN),^59^ GATv2,^60^ GraphSAGE^59^ and the directed message passing neural network (D-MPNN).^61^ All models were implemented using PyTorch Geometric^62^ and further details of the underlying graph theory and model architectural differences are in Section S2.

For each architecture, optimal hyperparameters for hyperparameter optimisation were identified using Optuna^63^ over 50 trials, with the full search spaces reported in Section S4. RedoxDB was split into training and validation sets in a 90 : 10 ratio using stratified sampling based on five quantile bins of *E*°. There is no overlap between RedoxDB and the experimental dataset, with no experimental molecules appearing in the training, validation, or test sets. Models were trained using the Adam optimiser, with MAE as the loss function and performance on the validation set was monitored using both the coefficient of determination (*R*^2^) and MAE. Early stopping with a patience of 30 epochs was applied to prevent overfitting, and the learning rate (LR) was adaptively adjusted using a ReduceLROnPlateau scheduler. Additional hyperparameters, including weight decay, dropout and random seed are listed in Section S4. A schematic overview of the pre-training workflow is shown in Fig. 4a.

**Fig. 4:**
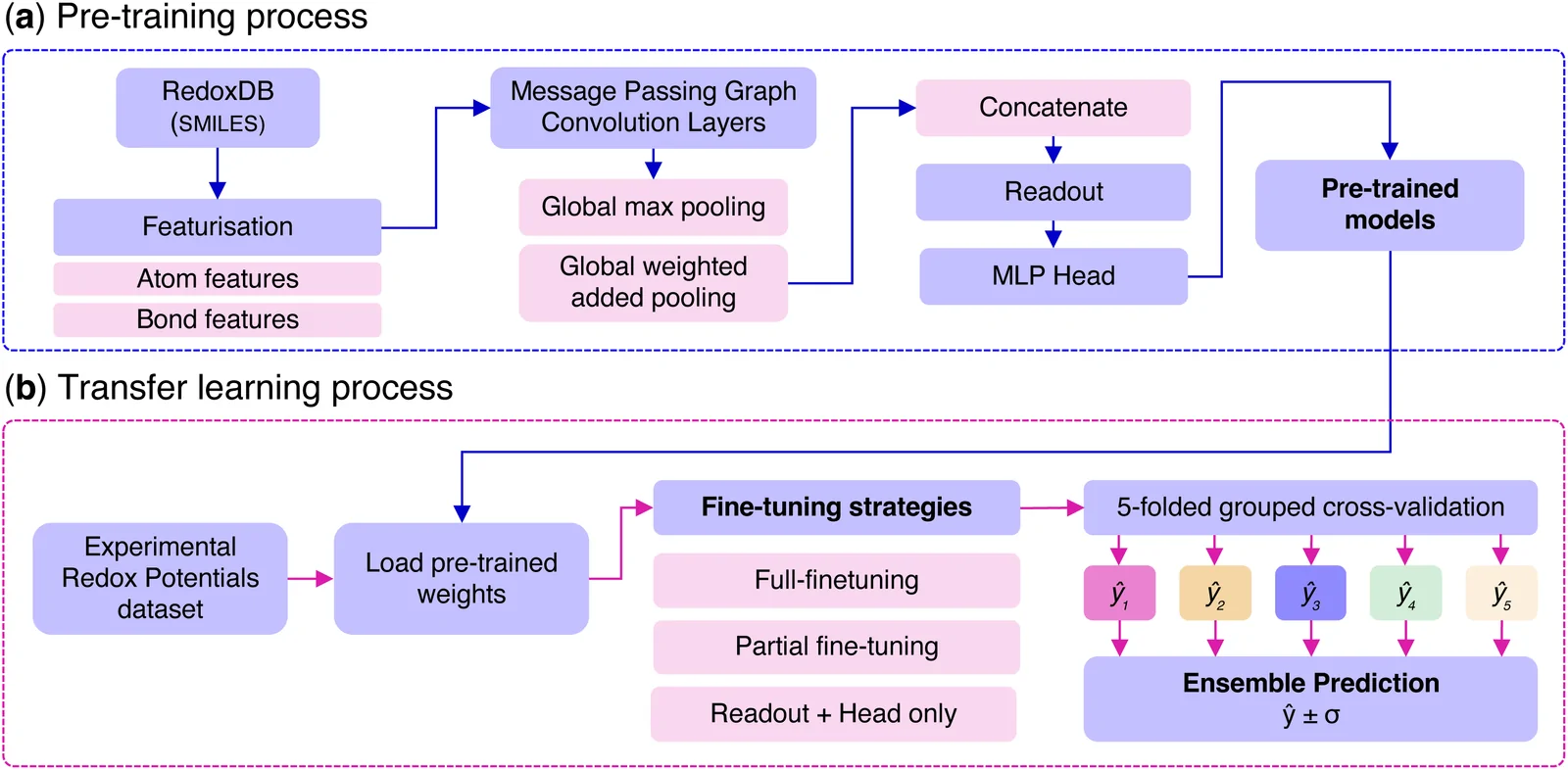
A schematic illustration of the RedEx workflow. (a) Pre-training pipeline for GNNs using the RedoxDB dataset. (b) Fine-tuning workflow, employing various fine-tuning strategies to optimise the pre-trained weights of the GNNs to the experimental *E*° dataset.

Following message passing, the atom-level embeddings were combined into a single graph-level representation using a sigmoid-weighted readout function as implemented by Ren *et al.*^64^ A learnable linear layer was first applied to each atom embedding to generate a scalar weight, which was passed through a sigmoid function to give an importance score between 0 and 1. These scores were used to form a weighted sum of the atom embeddings across the graph. In parallel, global max pooling was applied to retain the strongest signal from any individual atom. The final graph representation was obtained by concatenating the weighted-sum vector with the max-pooled vector and passed to the multi-layer perceptron (MLP) prediction head to generate a scalar prediction of *E*°. Compared to sum or mean pooling, this readout allows the models to place greater emphasis on atoms and chemical substructures dictating redox behaviour.

#### 2.3.2 Fine-tuning on experimental data

To bridge the domain gap between DFT-calculated and experimentally measured *E*° values, the pre-trained GNNs were fine-tuned on our high-fidelity curated experimental dataset as shown in Fig. 4b. Three fine-tuning strategies were evaluated relative to a no-pretraining baseline:

(1) Full fine-tuning: all pre-trained parameters were updated using the experimental dataset.

(2) Partial freezing: the lower 50% of the message-passing layers were frozen, while the upper message-passing layers, readout function and prediction head remained trainable.

(3) Head-only fine-tuning: all message-passing layers were frozen and only the graph-level readout and prediction head were updated.

The experimental dataset was used to train and evaluate all fine-tuning strategies. Data were split at the molecule level and stratified by chemical class, with molecule identity defined by canonicalised oxidised SMILES. All repeated experimental measurements of the same molecule were retained within the same partition. Approximately 20% of the unique molecules within each chemical class were assigned to the held-out test set, yielding an overall split of approximately 80 : 20, with small deviations due to the limited number of molecules in some classes. The remaining molecules were divided into five cross-validation folds using the same class-stratified, molecule-level grouping, ensuring that repeated measurements of the same molecule never occurred across training and validation folds. For each architecture and fine-tuning strategy, a second round of hyperparameter optimisation was performed, with the full search space and optimised configurations reported in Section S5. Retaining repeated measurements within the same fold enabled the model to capture the experimentally observed variability associated with each molecule.

This approach yielded five independently fine-tuned models for each GNN architecture and strategy, with each trained on a different subset of the experimental training data. For predictions on the unseen test set, the ensemble prediction of the 5 independent fold models was used. This approach both improves the robustness of the model due to the limited amount of experimental measurements and reduces sensitivity to any specific split.

Within each fold, models were trained for up to 200 epochs using the Adam optimiser on the MAE loss function. A ReduceLROnPlateau scheduler was used and early stopping was applied to prevent overfitting, with a patience of 30 epochs on the validation MAE. All runs use a random seed of 42. For GNNs with no-pretraining, the same HPO procedure and data-splitting approach was applied, but with random weights initialisation. In addition to GNNs, we evaluated the tabular baselines random forest,^65^ XGBoost^66^ and Catboost^67^ with the same data-splitting approach on the experimental dataset. The tabular baselines underperformed relative to the GNNs (Section S5.2), so were not pursued further in the main study.

#### 2.3.3 Evaluation metrics

Final model performance was assessed on the held-out test set (*n* = 36) using *R*^2^ and MAE. As a result of the grouping strategy, multiple experimental *E*° measurements for a single molecule exist in this test set. During inference, a single prediction was calculated using the mean ensemble prediction for each unique canonical SMILES and compared against the corresponding grouped experimental measurements. The model ensemble method allowed for uncertainty quantification, with the associated ensemble standard deviation used as a measure of model uncertainty.^68^ All GNN models were trained on a NVIDIA GeForce GTX 1050 Ti GPU with 4 GB VRAM.

## 3 Results and discussion

### 3.1 RedoxDB

With 57 core scaffolds and 27 substituent groups, RedoxDB constitutes, to our knowledge, the most structurally diverse database for aqueous organic redox-active molecules compared to existing publicly available datasets in this domain.^21^ The 7615 DFT-computed *E*° values span a total range of approximately −0.6 to +2.3 V relative to the SHE, as seen in Fig. 5a, covering the full region of practical interest for aqueous organic redox flow battery (AORFB) design of up to 1.229 V.

**Fig. 5:**
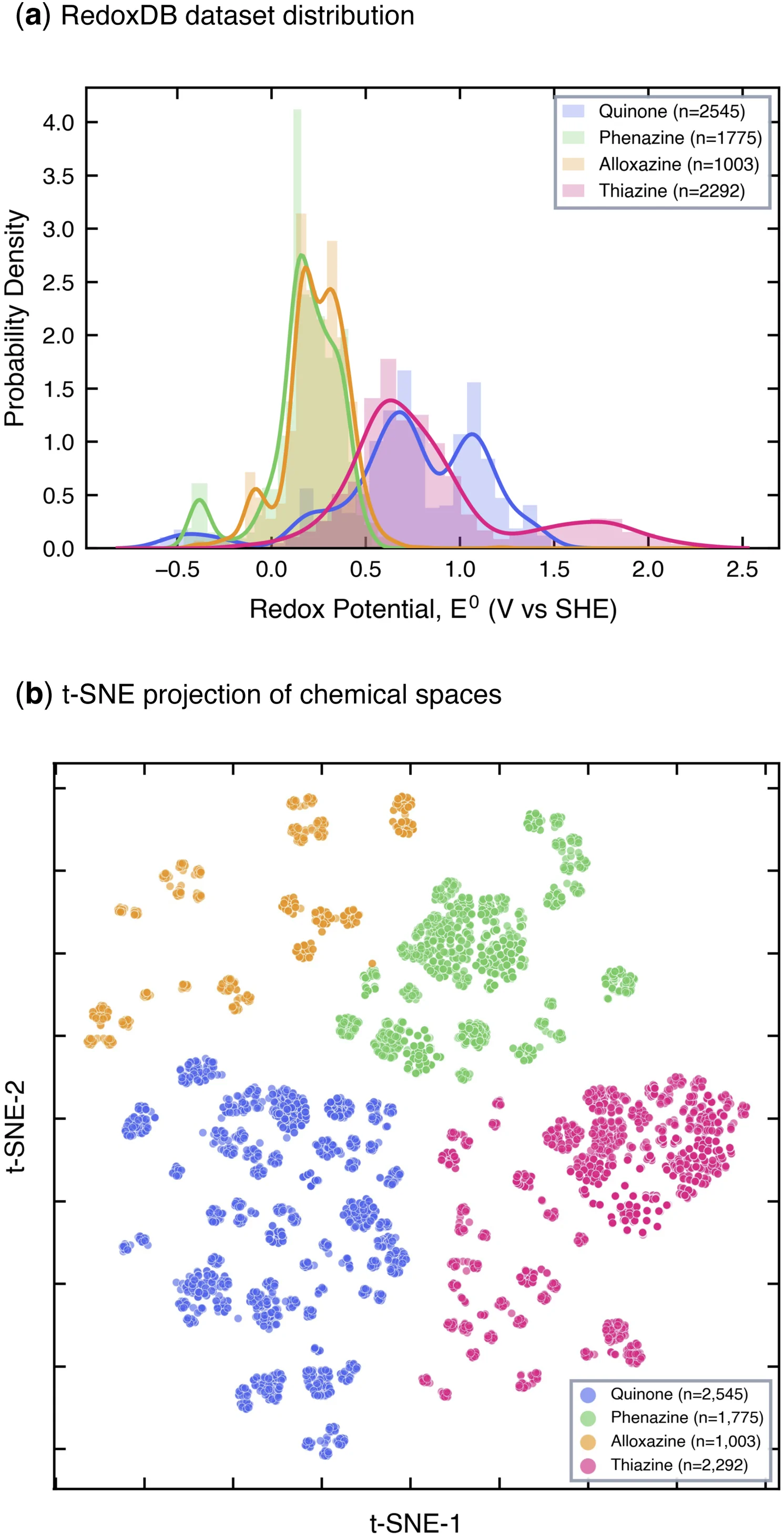
(a) Probability density plot of redox potentials in RedoxDB, classified by molecular class. (b) t-SNE embedding of Morgan fingerprints for the RedoxDB dataset (*n* = 7615), coloured by molecular class. The t-SNE highlights the structural diversity and distinct clusters of the quinones, phenazines, alloxazines and thiazines.

Clear scaffold specific trends are evident across this dataset. Thiazines (*n* = 2292) have the broadest and highest potential distributions, making them candidates for positive electrolytes, as seen in the literature.^31,69^ Quinones (*n* = 2545) encompass both negative and positive electrolyte candidates, while phenazines (*n* = 1775) and alloxazines (*n* = 1003) concentrate at lower potentials. These differences are also reflected in the t-SNE visualisation of chemical space as seen in Fig. 5b, where the four classes form largely distinct clusters.

### 3.2 Pre-trained model performance

All five GNN architectures demonstrated strong predictive performance when pre-trained on RedoxDB (Table 1), with validation MAEs ranging from 0.036–0.077 V and *R*^2^ values between 0.946–0.983. This indicates that the models successfully learned the underlying structure–property relationships governing DFT-computed *E*° values directly from molecular graphs. Among the tested models, GIN achieved the lowest validation MAE (0.036 V, *R*^2^ = 0.983), followed closely by GATv2 (0.040 V, *R*^2^ = 0.979), GraphSAGE (0.044 V, *R*^2^ = 0.977), and D-MPNN (0.046 V, *R*^2^ = 0.975), while GCN showed comparatively lower performance (0.077 V, *R*^2^ = 0.946).

**Table 1:** Computational pre-training performance on RedoxDB (90/10 split, *n* = 7575). Models are arranged in order of decreasing MAE

Model	Params	MAE (V)	*R* ^2^
GCN	101.5 K	0.0767	0.946
D-MPNN	77.4 K	0.0460	0.975
GraphSAGE	59.7 K	0.0438	0.977
GATv2	925.2 K	0.0404	0.979
GIN	167.6 K	0.0361	0.983

#### 3.2.1 Quantifying the domain gap

To quantify the domain gap between DFT-derived and experimentally measured *E*°, we evaluated the zero-shot transfer performance of the pre-trained GNNs on the curated experimental test set (*n* = 36 unique molecules), without any fine-tuning. The underlying experimental dataset comprises 264 measurements (*n* = 236 unique molecules) drawn from the literature, dominated by quinones (*n* = 202), with smaller numbers of phenazines (*n* = 35), alloxazines (*n* = 20) and thiazines (*n* = 7).

The results are summarised in Table 2, with the corresponding parity plots shown in Fig. 6. Although the MAEs remain within the broad error range typically associated with DFT-level redox potential calculations, the *R*^2^ values are uniformly poor and in several cases, strongly negative, indicating that models trained solely on DFT-derived data do not transfer reliably to the experimental domain.

**Table 2:** Zero-shot transfer performance of pre-trained models evaluated directly on the experimental test set without fine-tuning (*n* = 36 molecules), sorted by decreasing MAE

Model	MAE (V)	*R* ^2^
GIN	0.2883	−2.505
GATv2	0.2376	−1.435
GraphSAGE	0.2364	−1.168
D-MPNN	0.2262	−0.552
GCN	0.1900	−0.333

**Fig. 6:**
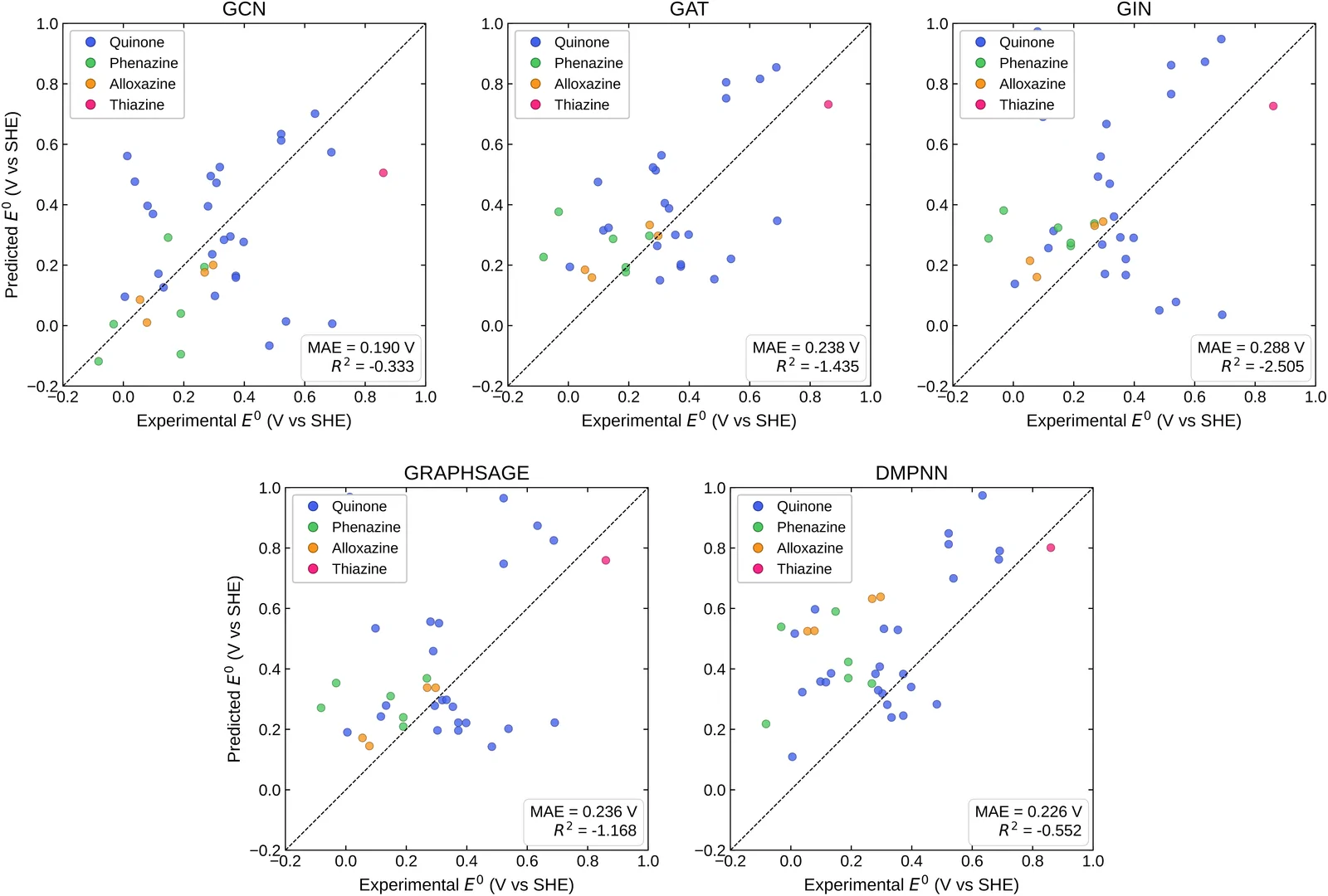
Zero-shot transfer performance of the pre-trained GNNs on the held-out experimental test set (*n* = 36 unique molecules). Predictions are shown without any fine-tuning on experimental data, with points coloured by molecular class and the dashed line indicating perfect agreement. Across all architectures, substantial scatter and weak agreement with the parity line are observed, highlighting the computational–experimental domain gap.

The parity plots give a clearer picture of the nature of the domain gap. All five models show considerable scatter and weak agreement across the full experimental window. The zero-shot MAEs span 0.190–0.288 V, with uniformly poor *R*^2^ values ranging from −0.333 to −2.505. GCN performs least poorly in this setting (MAE = 0.190 V, *R*^2^ = −0.333), whereas GIN performs worst (MAE = 0.288 V, *R*^2^ = −2.505). These results show that models trained solely on DFT-derived data do not transfer reliably to the experimental domain without fine-tuning.

The DFT-experimental domain gap was further examined by directly comparing the DFT-calculated redox potentials with the experimental measurements in Section S1.6.1. The effect of the correction varied between the chemical classes, with little change for quinones (MAE = 0.191 → 0.185 V) but larger improvements for phenazines (MAE = 0.180 → 0.117 V) and alloxazines (MAE = 0.062 → 0.025 V). For thiazines, the MAE decreased from 0.123 to 0.096 V, although only seven measurements were available. Taken together, these results show that the computational–experimental domain gap is substantial and cannot be overcome by direct application of DFT-trained models alone, thereby justifying the use of transfer learning to adapt the learned representations to the experimental domain.

The origin of the domain gap is driven by multiple contributing factors. DFT calculations are subject to systematic errors, arising from the choice of functional, basis set, solvation model and thermodynamic assumptions, particularly for PCET reactions. Solvation is especially challenging to model accurately, as it is highly sensitive to the solvent environment and accompanying structural re-organisation.^70^ Implicit solvation models such as SMD treat solvents as a continuum, which captures broad solvation trends but not all molecular specific interactions such as hydrogen bonding.^71–73^ Molecular dynamics (MD) has been employed for explicit solvation modelling, but has a greater computational demand.^74^

In addition, experimental *E*° values compiled from the literature contain unavoidable variability associated with difference in electrolyte composition, measurement protocol, instrumentation and post-processing across research laboratories.^75^ Collectively, these factors make the domain gap more complex than linear calibrations and motivate our approach of transfer learning to adapt pre-trained DFT representations to the experimental domain.

### 3.3 Transfer learning performance

Following the 80 : 20 splitting strategy described in Section 2.3.2, a stratified test set of 50 measurements (36 unique molecules) was held out, and the remaining 214 measurements were used for the 5-fold cross-validated fine-tuning of the pre-trained GNNs. Table 3 summarises the transfer learning performance of the 5 GNN architectures across all fine-tuning strategies on the held out experimental test set. The results show that fine-tuning improved predictive performance relative to the no-pretraining baselines, demonstrating that GNNs initialised from pre-trained checkpoints transferred more effectively to the experimental domain than their randomly initialised counterparts.

**Table 3:** Transfer learning performance across fine-tuning strategies on the experimental test set (*n* = 36 unique molecules). Bold indicates the best combination of MAE and *R*^2^ of all models and strategies. Uncertainty estimates are given in Section S5.4

Model	No pre-training		Full fine-tuning		Partial fine-tuning		Readout + head	
	MAE (V)	*R* ^2^	MAE (V)	*R* ^2^	MAE (V)	*R* ^2^	MAE (V)	*R* ^2^
GCN	0.0697	0.831	0.0619	0.874	0.0644	0.868	0.0795	0.776
GATv2	0.0750	0.816	0.0590	0.887	**0.0567**	**0.900**	0.0869	0.708
GIN	0.0896	0.720	0.0562	0.888	0.0603	0.874	0.0627	0.863
GraphSAGE	0.0756	0.825	0.0596	0.875	0.0740	0.806	0.0748	0.813
D-MPNN	0.0947	0.663	0.0757	0.790	0.0671	0.832	0.0604	0.857

The optimal fine-tuning strategy depended on the architecture, with full-tuning yielding the best performance for GCN, GIN and GraphSage, whereas the readout + head strategy performed best for D-MPNN. Overall, GATv2 with partial fine-tuning delivered the strongest performance, achieving a test MAE of 0.057 V and *R*^2^ of 0.900, a 24% improvement for MAE and 10% for *R*^2^ over the GATv2 baseline (MAE = 0.075 V, *R*^2^ = 0.816). This result identified GATv2 as the strongest performing architecture in the transfer learning setting and motivated its selection as the final RedEx model. Although the difference between full and partial fine-tuning for GATv2 was modest, the slight improvement with partial fine-tuning suggests that freezing the lower message-passing layers helped to preserve transferable chemical information learned from RedoxDB, while the prediction head and upper layers adapted to the new domain. By contrast, the generally weaker performance of the readout + head fine-tuning for most architectures indicate that this strategy is too restrictive for excellent adaptation.

Fig. 7 presents the parity plot for RedEx on the held-out experimental test set. The ensemble predictions show strong agreement with experiment, with an *R*^2^ = 0.900 and a MAE = 0.057 V. Most predictions lie closely around the parity line, indicating that the model can capture the important structure–property relationships in the experimental domain. Although only limited class-wise comparison can be drawn from the MAE values due to the scarcity of phenazines, alloxazines and especially thiazines in the test set, inspecting the individual predictions for these classes, indicates that the model adapted well to the new domain despite a small training set.

**Fig. 7:**
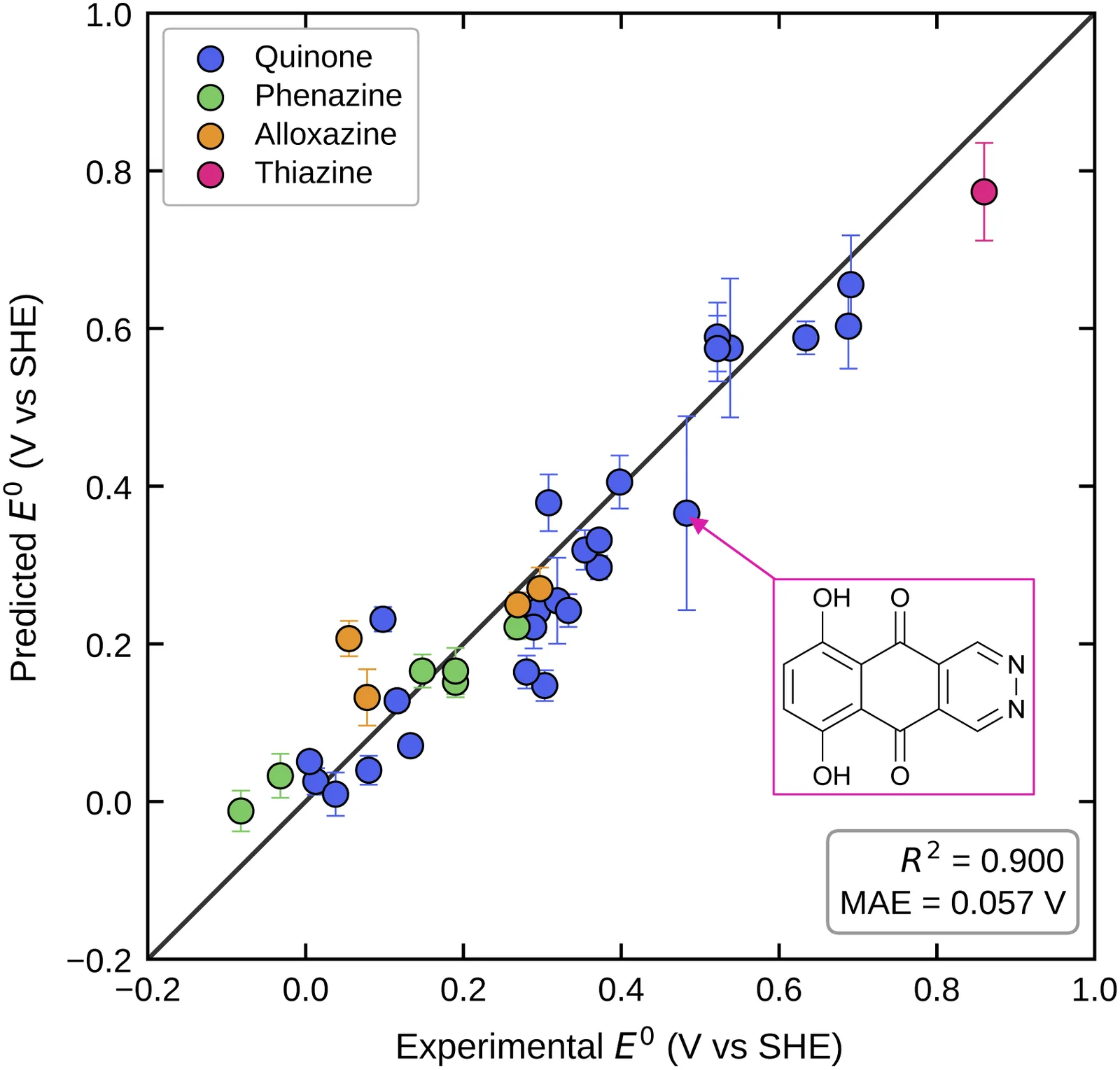
Parity plot of RedEx predictions on the unseen experimental test set (*n* = 36). Predicted *E*° values are shown against experimental *E*° values, with points coloured by molecular class. Error bars represent the model uncertainty derived from the 5-model ensemble and the solid line denotes perfect agreement. RedEx achieves an *R*^2^ = 0.900 and an MAE = 0.057 V. The prediction with the highest uncertainty is labelled with the corresponding molecular structure of 2,3-diaza-5,8-dihydroxy-anthracene-9,10-dione.

The phenazine and alloxazine predictions in particular remain close to experimental values, suggesting that the transfer learned representations can retain information even for under-represented classes. By contrast, the experimental test set has only a single thiazine value, therefore no strong conclusion can be drawn regarding class-specific performance for the ML prediction of that system. Quinones, however, are sufficiently represented and show accurate predictions across a wide range of *E*°, demonstrating that RedEx is able to capture the influence of both scaffold structure and FG substitution patterns.

#### 3.3.1 Uncertainty quantification

A practical advantage of RedEx is the five-fold ensemble, which provides an estimate of epistemic uncertainty.^68^ In general, larger predictive uncertainties are associated with molecules occupying more weakly represented regions of the learned chemical space. Here, predictive uncertainty was quantified as the standard deviation across the five fold-specific ensemble predictions. For RedEx, the mean predictive uncertainty on the test set was 0.0391 V, indicating relatively close agreement between ensemble members and therefore comparatively high confidence for most unseen molecules.

The largest outlier was observed for the 2,3-diaza-5,8-dihydroxy-anthracene-9,10-dione highlighted in Fig. 7, with a *E*° prediction of 0.366 ± 0.123 V and a MAE of 0.117 V.^76^ This pyridazine-fused quinone scaffold is absent from both the experimental training data and RedoxDB. The introduction of heteroatoms into the fused ring system of anthraquinone can alter the *E*° significantly, as reflected by the distinct behaviour of different ORM classes, and such effects are difficult to infer reliably when closely related motifs are not represented. Despite this, the model still returns a prediction for 2,3-diaza-5,8-dihydroxy-anthracene-9,10-dione within the broad error range typically associated with DFT-level *E*° calculations. This suggests that RedEx has learnt transferable structure–property relationships that enable accurate prediction of unseen molecules within or close to the chemical space represented during training, as all other test set predictions are also within this range. This distinction is important in the context of ORM design. Predicting the *E*° of completely unseen structural motifs remains inherently challenging, especially when the underlying redox chemistry differs from that represented in the training set.^28^ However, electrolyte discovery is more often an iterative optimisation problem, centred on known scaffold families and local functionalisations. In this setting, RedEx offers a practical advantage by enabling the rapid prediction of experimental *E*° values for candidates within or near the learned chemical space. RedEx can be used as a screening tool that is significantly faster than either CV experiments or DFT calculations, and could be refined further with additional DFT or experimental data.

### 3.4 Interpretability

To examine the learned representation space, we firstly projected the graph-level embeddings of RedEx using t-SNE, as shown in Fig. 8. Although t-SNE is primarily a qualitative visualisation, the resulting embeddings show the four ORM classes in latent space. Quinones occupy the broadest region, consistent with the greater structural diversity and broader redox potential range. The held-out test set molecules lie adjacent or within the training set regions rather than forming isolated clusters, indicating the model has learned chemically coherent latent representations of aqueous ORMs.

**Fig. 8:**
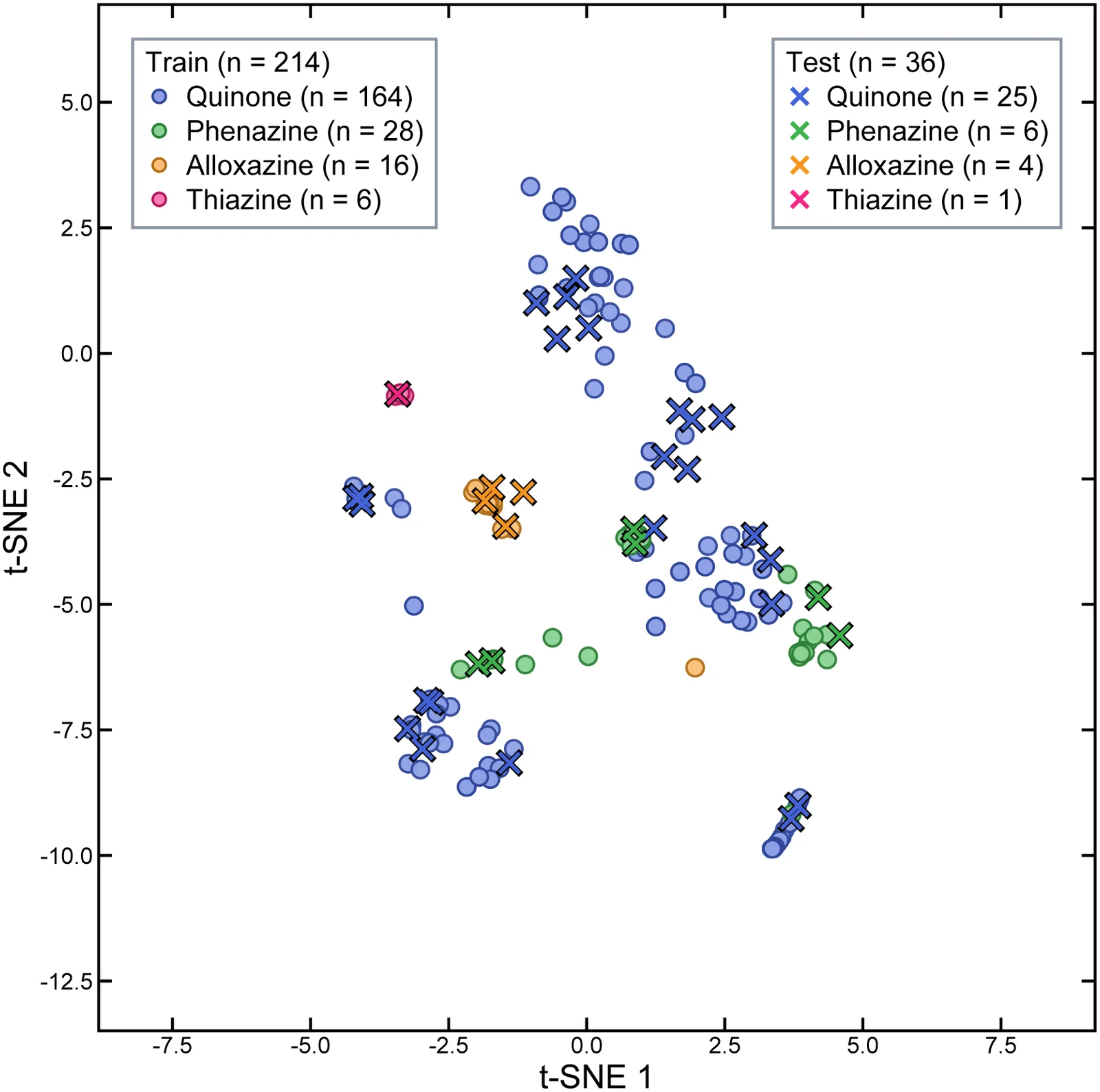
t-SNE projection of the graph-level embeddings learnt by RedEx, with the held-out experimental test set molecules superimposed on the training data chemical space. Colours denote ORM class and crosses indicate test molecules.

A further advantage of RedEx is that, by employing a GATv2 architecture, it enables the extraction of atom-level attention weights and therefore qualitative interpretation of the contribution of chemical motifs to the predicted *E*°. To probe the chemical plausibility of the learned representations of RedEx, attention maps were extracted for representative molecules spanning all four ORM classes, including molecules with the highest and lowest *E*°.

Across all examples, the highest attention is often assigned to chemically meaningful sub-structures, including redox-active centres and key substituents which are known to influence the *E*°. Quinone examples (Fig. 9a and b) had the strongest attention concentrated on the carbonyl oxygens, indicating the model can recognise the quinone redox-active centres. Fig. 9b is a benzoquinone-type system, which is consistent with its more positive *E*° relative to AQDBe (Fig. 9a).^77^ The attention pattern for this molecule also suggests that RedEx can capture the electronic balance imposed by the substituents. The nitrogens in the fused ring are EWGs whereas the methyl groups are EDGs, offsetting any major increases in *E*°.

**Fig. 9:**
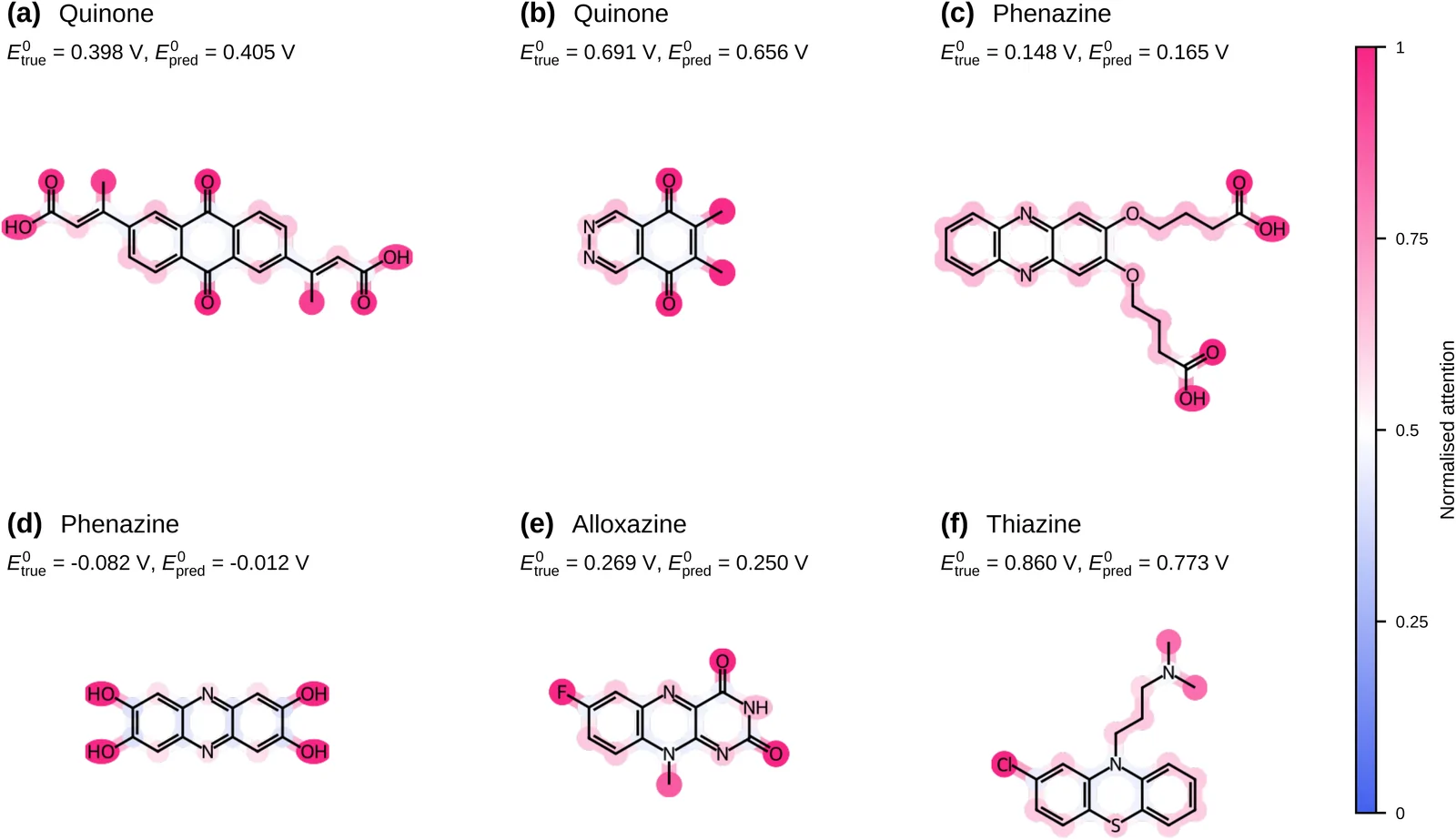
Attention heat maps extracted from RedEx, highlighting chemically relevant motifs contributing towards experimental *E*° prediction for (a) AQDBe, (b) 6,7-dimethylphthalazine-5,8-dione, (c) DAEP, (d) 1,2,7,8-tetrahydroxyphenazine, (e) chlorpromazine, (f) 7-fluoro-10-methylalloxazine.^31,42–44,49,78^ The predicted values and the experimental *E*° values and system class are provided for each example. The colour scale indicates normalised attention weight, with pink denoting higher attention and therefore a greater contribution to the prediction, and blue denoting lower attention.

For the phenazine examples (Fig. 9c and d), attention is distributed across both the phenazine core and the four hydroxyl substituents. The highest attention weight is applied to the hydroxyl substituents which is chemically consistent, as hydroxyl substituents are strong EDGs that shift the *E*° to more negative values. The attention pattern therefore suggests that RedEx can capture the importance of substituent effects in lowering the *E*° of phenazine derivatives.

The chlorpromazine example (Fig. 9e) is a particularly interesting case because thiazines are sparsely represented in the test-set. Experimentally, *N*-substituted phenothiazines are known to exhibit comparatively higher redox potentials relative to the unsubstituted phenothiazine core and RedEx captures this broad trend, although it under-predicts the experimental value.^31^ The attention map assigns attention to the phenothiazine core and chloro substituent, both of which are chemically relevant to the redox potential. Attention is also observed on the tertiary amine side chain, which is not expected to participate directly in the redox mechanism.

This attention may reflect indirect information carried by the side chain, including *N*-substitution, charge distribution or similarity to higher *E*° values from structures in the training data. More broadly, the curated experimental literature may contain selection biases towards molecules that combine high *E*° with a higher aqueous solubility. Ionisable groups such as amines are frequently introduced to improve solubility and may occur more often among high-potential molecules in experimental literature. The model may consequently learn an association between these solubilising motifs and higher *E*°, even when the groups themselves do not directly control the redox process. This illustrates the qualitative nature of the attention analysis; attention weights reveal features deemed important by the model but they do not necessarily correspond to mechanistic redox sub-structures.

Fig. 9f shows that the fused *N*-containing heterocyclic core has been identified as the redox-active centre, while the highest attention has been assigned to the methyl and fluoro substituents. This is broadly consistent with the electronic roles of these groups; the methyl group is an EDG and the fluorine is an EWG, so their effects partially counterbalance, with a resulting prediction dominated by the alloxazine scaffold, which is generally associated with a lower *E*°.

Taken all together, the attention maps suggest that RedEx captures chemically meaningful relationships involving both substituent-driven electronic effects and broader molecular structure for aqueous ORMS.

## 4 Conclusions

The redox potential (*E*°) is a defining property of aqueous organic electrolytes, as it determines whether charge storage can proceed within the electrochemical stability window of water. Accurate prediction of *E*° is therefore central to the accelerated design of new ORMS for AORFBs. Here, we present three contributions to this goal. First, we introduce RedoxDB, a new, open-source computational dataset comprising 7615 DFT-calculated *E*° values, spanning the major ORM classes of quinones, phenazines, alloxazines and thiazines, generated using an automated high-throughput workflow. Secondly, we curate dataset of 264 experimental redox potentials from the literature. Finally, we developed RedEx, an ensemble GNN trained with transfer learning for the rapid prediction of experimental aqueous *E*° values.

Pre-training on RedoxDB enabled GNN models to learn chemically meaningful structure–property relationships at the DFT level, with strong performance across all architectures. However, zero-shot evaluation on experimental data revealed a substantial computational–experimental domain gap. Transfer learning was shown to significantly reduce this gap; fine-tuning the pre-trained GNNs on experimental data consistently improved prediction of experimental *E*° values relative to a no-pretraining baseline.

Among the architectures studied, the ensemble GATv2 with partial fine-tuning achieved the best overall performance, achieving a MAE of 0.057 V and an *R*^2^ of 0.900 on a held-out test set and was selected as the RedEx architecture. RedEx provides both an estimate of epistemic uncertainty from its ensemble framework and interpretability *via* the extraction of attention weight maps. Interpretability analysis revealed that RedEx captured chemically meaningful intuition, assigning strong attention to chemical motifs known to dictate the *E*°.

RedEx is intended to be used as an iterative design tool for exploring ORM candidates within, or close to the learned chemical space represented by RedoxDB. While the model may provide predictions for more distant OOD structures, these should be interpreted with caution, particularly where the underlying redox mechanism is not represented in the training data. Accordingly, we do not position RedEx as a general virtual-screening model but rather as a targeted tool for iterative structural design around known redox-active scaffold families.

The principal limitation of RedEx is the size and composition of the experimental dataset, strongly biased towards quinones, and limited in size (*n* = 264). Despite strong predictive performance for alloxazines, phenazines and thiazines, the confidence of predictions is much greater for quinones. Expanding both datasets to include broader redox chemistries, such as viologens, TEMPO derivatives and thiazine dyes, would improve both the model confidence and generalisation.^11,79^ The current scope is also limited by the use of a single predefined redox transformation for each scaffold class, with RedEx using only the reactant molecular structure as input and therefore unable to distinguish between multiple possible redox processes for the same molecule.

Another limitation is that RedEx is trained as a regression model and therefore cannot distinguish redox-active, reversible molecules from unstable candidates. Incorporating counter-examples, such as redox cores known to be electrochemically irreversible or unstable, would enable future classification-regression workflows and broaden the applicability of RedEx for virtual screening. The inclusion of more experimentally tested candidates that were discarded during the earlier stages of electrolyte development, for example because of insufficient solubility could also help reduce the selection bias inherent in datasets derived primarily from successful literature reports. Future development could therefore combine uncertainty-guided active learning with targeted experimental or computational data acquisition.

Advances in machine-learned force fields (MLFFs) provide another route for extending this work. Chen *et al.* recently demonstrated that large-scale pre-trained models such as MACE-Omol and UMA can accelerate components of *E*° calculations, although DFT single-point energy corrections remain important for accurate predictions.^20^ Such models could facilitate the expansion of RedoxDB and provide an additional pre-training stage prior to RedoxDB or experimental fine-tuning, representing an interesting direction of future work.

Overall, this work demonstrates that GNNs, when combined with transfer learning, can reliably predict *E*° across computational and experimental domains, effectively closing the domain gap. RedoxDB and RedEx provide useful tools for accelerating the design of new ORMS for AORFBs. The datasets and code are openly available at https://github.com/datmdoan/RedEx.

## Author contributions

D. M. D. designed the workflows, conducted experiments and analysed all results. Q. S. and A. K. contributed to project conceptualisation. K. E. J. supervised the project. D. M. D. drafted the manuscript and all authors contributed to the final version.

## Conflicts of interest

There are no conflicts of interest to declare.

## Supplementary Material

SC-OLF-D6SC05970H-s001

## Data Availability

The data and code supporting this study are openly available on GitHub. The RedEx repository (https://github.com/datmdoan/RedEx, 10.5281/zenodo.21314449) contains the transfer-learning workflow, the RedEx model and the datasets used in this work. In particular, RedoxDB.csv contains the computational dataset, including oxidised and reduced SMILES strings, DFT-calculated redox potentials, and associated electronic and thermodynamic properties such as solvation free energies, HOMO energies, and LUMO energies. Experimental_redox_potentials.csv contains the curated experimental dataset together with the corresponding literature sources. Custom tools used for scaffold enumeration and generation of reduced SMILES strings are available at https://github.com/datmdoan/compchem-tools, https://doi.org/10.5281/zenodo.21314460. Supplementary information (SI): RedoxDB scaffolds, DFT benchmarking and hyperparameter optimisation. See DOI: https://doi.org/10.1039/d6sc05970h.
