# Supplementary material for: RedoxDB and RedEx: closing the DFT-experiment domain gap in redox potential prediction with transfer learning
Source: Chem Sci. 2026 Oct 15. Online ahead of print. doi: 10.1039/d6sc05970h (PMC13686153; doi:10.1039/d6sc05970h)
Supplement: Supplementary file 1 [file SC-OLF-D6SC05970H-s001.pdf]

Supplementary Information (SI) for Chemical Science.
This journal is © The Royal Society of Chemistry

Supporting Information: RedoxDB and RedEx: closing the DFT-experiment domain gap in redox potential prediction with transfer learning

Dat M. Doan, Qilei Song, Anthony Kucernak and Kim E. Jelfs

Department of Chemistry, Molecular Sciences Research Hub, White City Campus, Imperial College London, London, W12 0BZ, UK.
Department of Chemical Engineering, South Kensington Campus, Imperial College London, SW7 2AZ, UK.

E-mail: k.jelfs@imperial.ac.uk

Contents

S1 RedoxDB Scaffolds **3**
S1.1 Quinones . . . 3
S1.2 Phenazines . . . 4
S1.3 Alloxazines . . . 5
S1.4 Thiazines . . . 6
S1.5 Functionalisations . . . 7
S1.6 Benchmarking DFT calculations . . . 8
S1.6.1 Direct comparison of DFT-calculated and experimental redox potentials . . . 8

S2 Graph neural network architecture **11**

S3 Featurisation **12**

S4 Pre-training **13**
S4.1 Pre-training Hyperparameter Optimisation . . . 13

S5 Fine-tuning **14**
S5.1 Fine-tuning HPO . . . 14
S5.2 Tabular baselines . . . 15
S5.3 Experimental test set results . . . 16
S5.4 Fine-tuning strategy ablation . . . 16

S1. RedoxDB Scaffolds

S1.1 Quinones

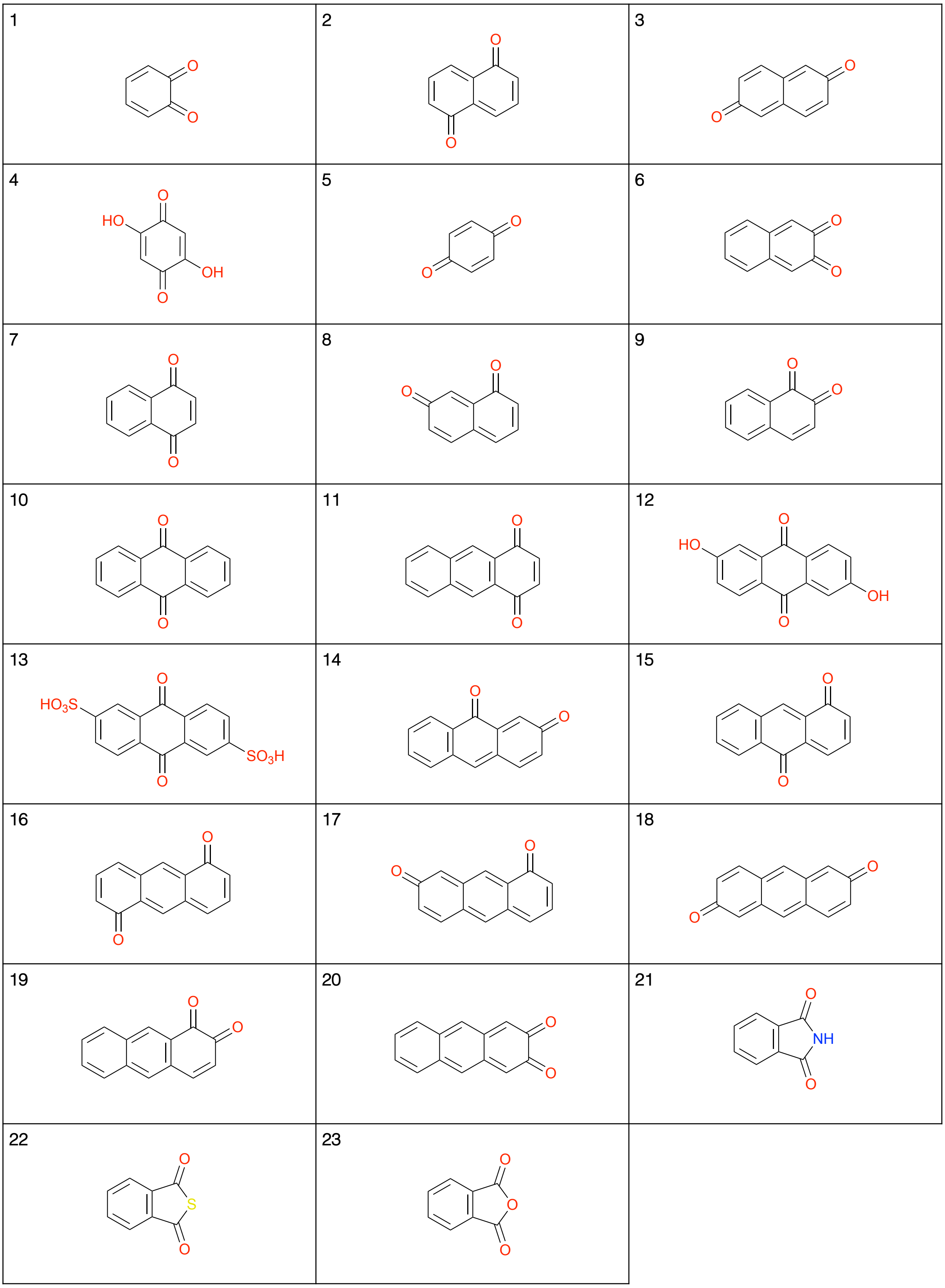

Figure S1: The 23 selected quinone core structures for enumeration in RedoxDB.

S1.2 Phenazines

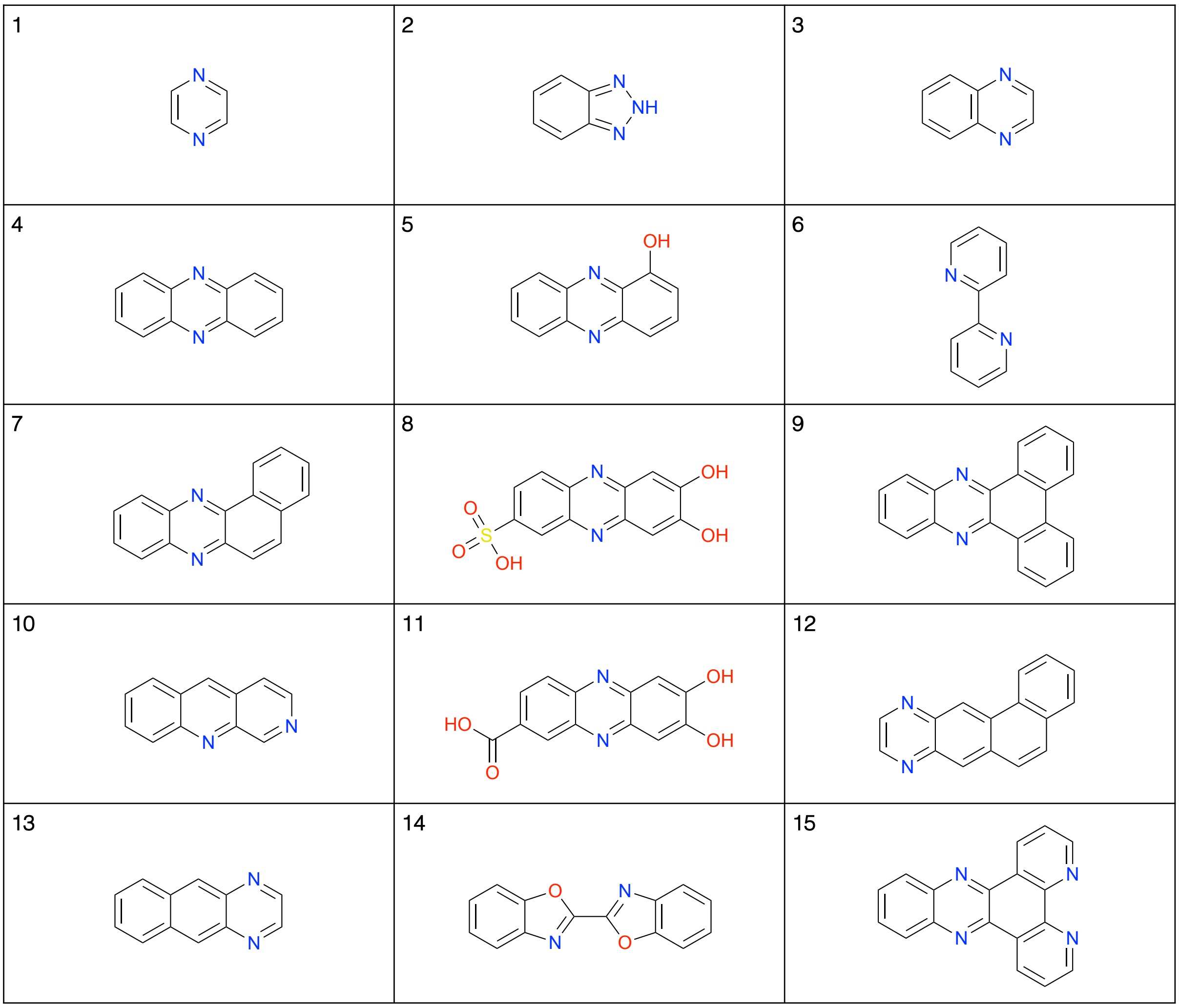

Figure S2: The 15 selected phenazine core structures for enumeration in RedoxDB.

S1.3 Alloxazines

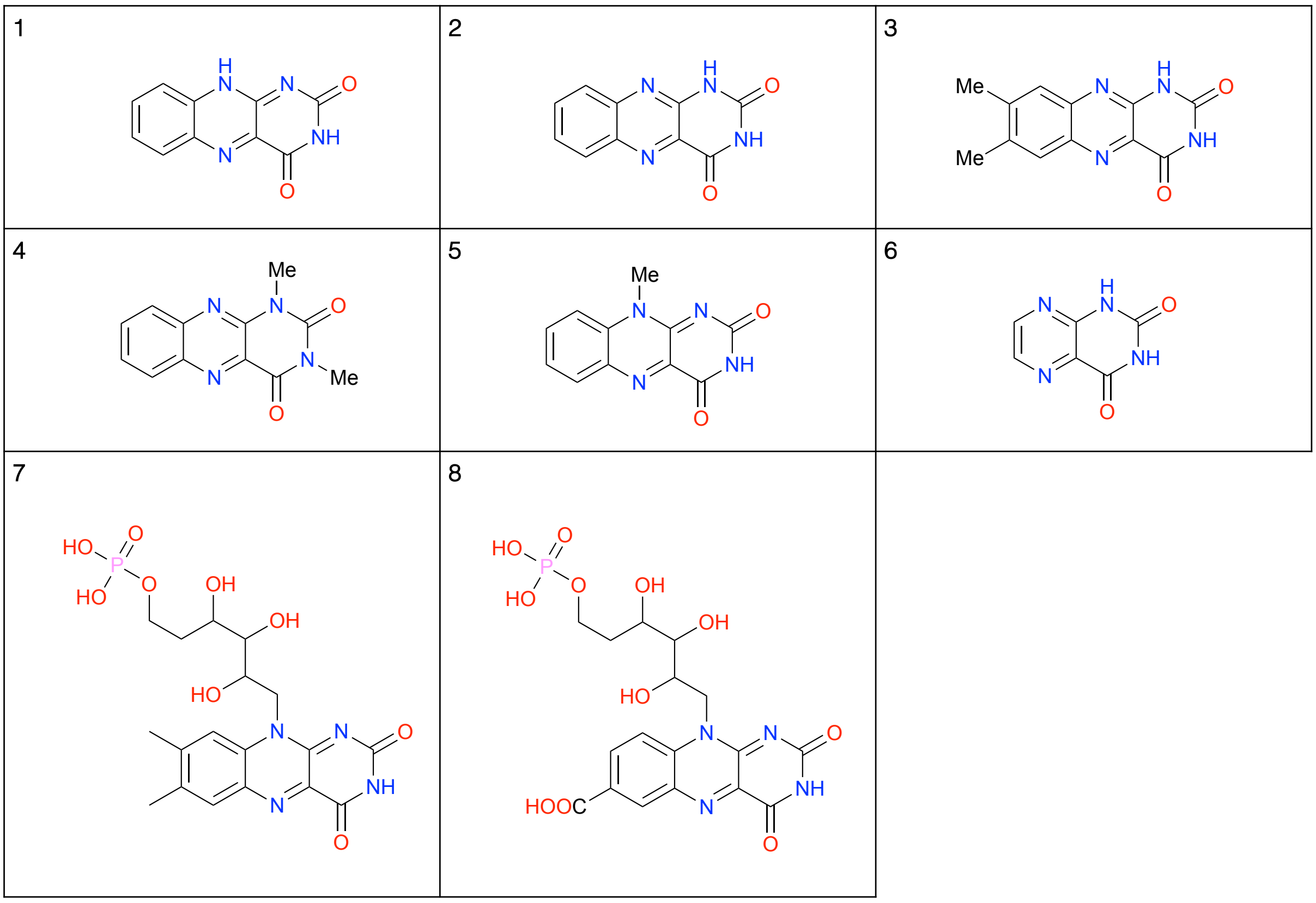

Figure S3: The 8 selected alloxazine core structures for enumeration in RedoxDB.

S1.4 Thiazines

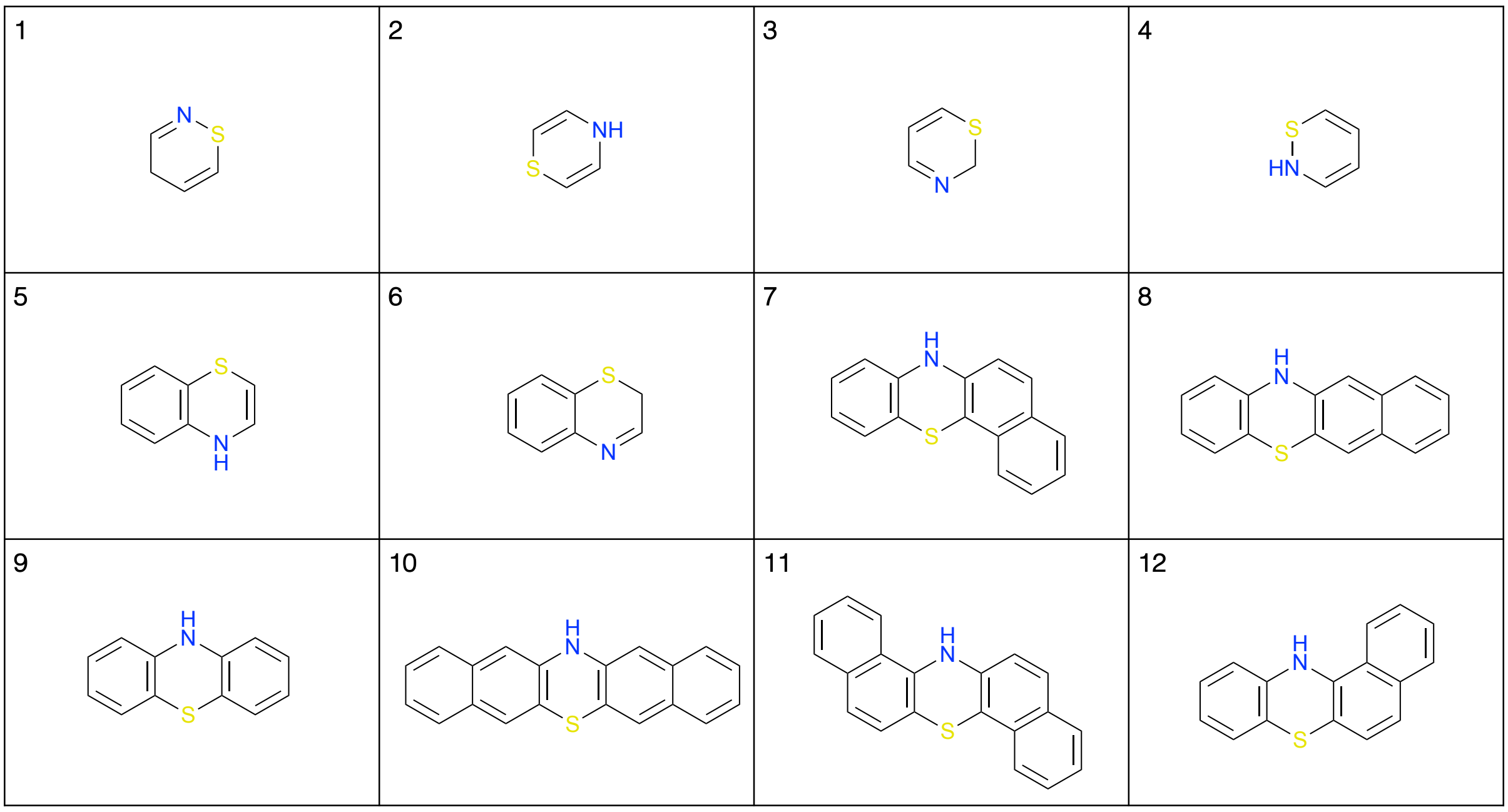

Figure S4: The 12 selected thiazine core structures for enumeration in RedoxDB.

Thiazine dyes such as methylene blue are well established and experimentally stable redox-active molecules, but were excluded from both RedoxDB and the experimental dataset because their redox chemistry is not directly comparable to the thiazine derivatives modelled in this work.[1] Their complex thermodynamic cycles could not be modelled consistently within the automated density functional theory (DFT) workflow and were therefore excluded from both computational and experimental datasets to avoid introducing non-comparable labels. RedEx is therefore most reliable for redox chemistries represented in the training data.

S1.5 Functionalisations

A total of 27 functional groups were used for combinatorial enumeration across the 57 scaffolds: –F, –Cl, –Br, –OH, $–NH_2$, $–N(CH_3)H$, $–N(CH_3)_2$, $–OCH_3$, $–OCH_2CH_3$, $–CH_3$, $–CH_2CH_3$, $–C_2H_3$, $–C_6H_5$, –SH, $–SCH_3$, $–SiH_3$, $–NO_2$, –CN, –C(=O)H, $–C(=O)CH_3$, $–C(=O)OCH_3$, $–CF_3$, $–SO_3H$, $–SO_3^-$, $–PO_3H_2$, $–P(CH_3)_2$, $–NHCH_2CH_2COOH$.

S1.6 Benchmarking DFT calculations

The DFT methodology used in this work was benchmarked against experimental redox potentials for representative compound classes. Table **S1** compares the mean absolute error (MAE) of the B3LYP-D3 and M06-2X functionals for each scaffold family. The same basis set of 6-31++G(d,p) is used.

Table S1: Validation of DFT-computed redox potentials against experimental values for representative compound classes.

Compound class	B3LYP-D3 MAE (V)	M06-2X MAE (V)
Quinones	0.24	0.19
Phenazines	0.21	0.18
Thiazines	0.37	0.12
Alloxazines	0.12	0.06

S1.6.1 Direct comparison of DFT-calculated and experimental redox potentials

The M06-2X DFT-calculated redox potentials were compared directly with the corresponding experimental measurements. All 264 experimental values were retained, including repeat measurements for the same molecule.

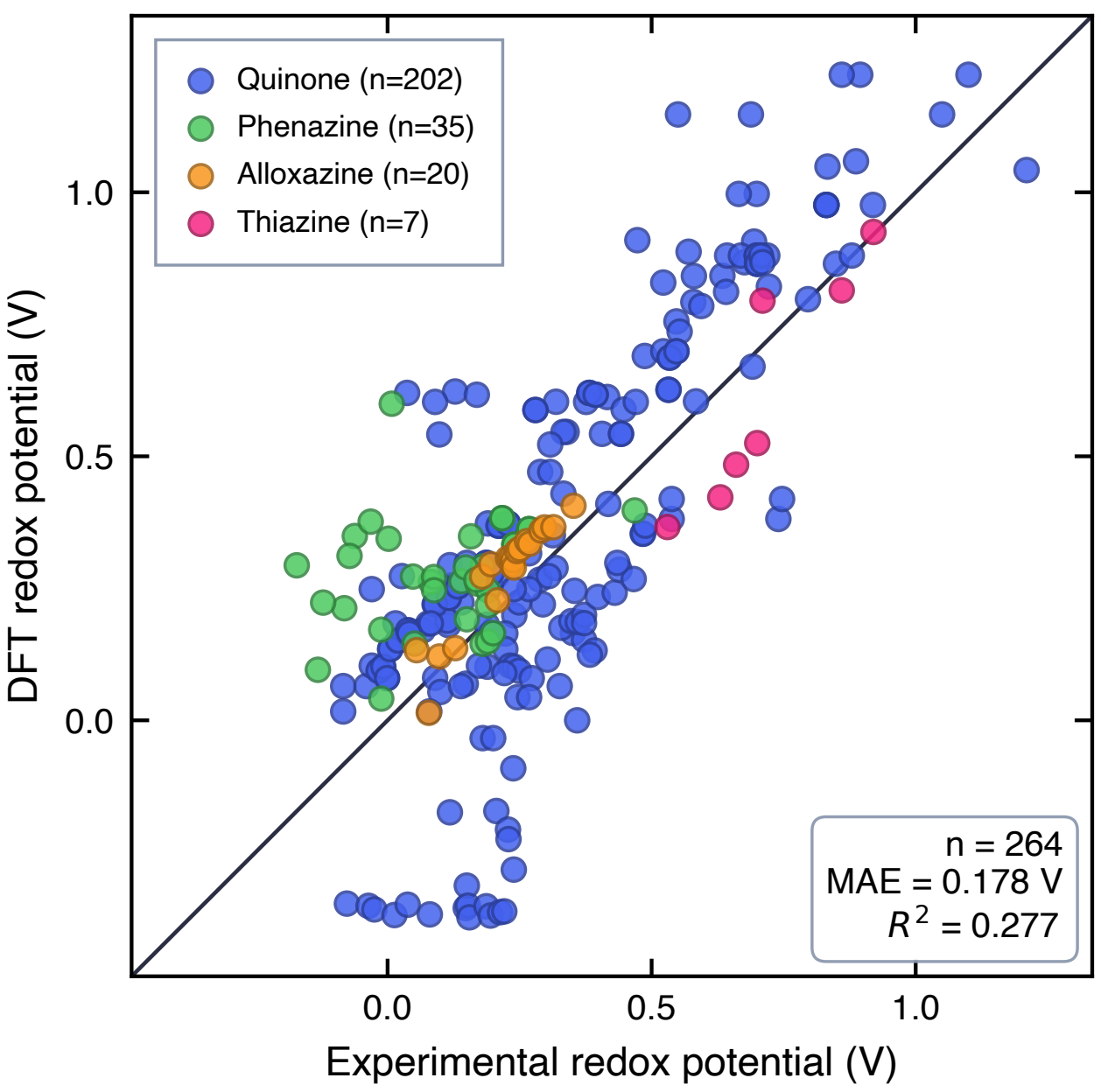

Figure S5: Direct comparison between M06-2X DFT-calculated and experimentally measured redox potentials for the complete experimental dataset. The diagonal line represents perfect agreement between calculated and experimental redox potentials. All 264 experimental measurements are retained, including repeated measurements of the same molecule, to preserve the experimentally observed variability in reported redox potentials.

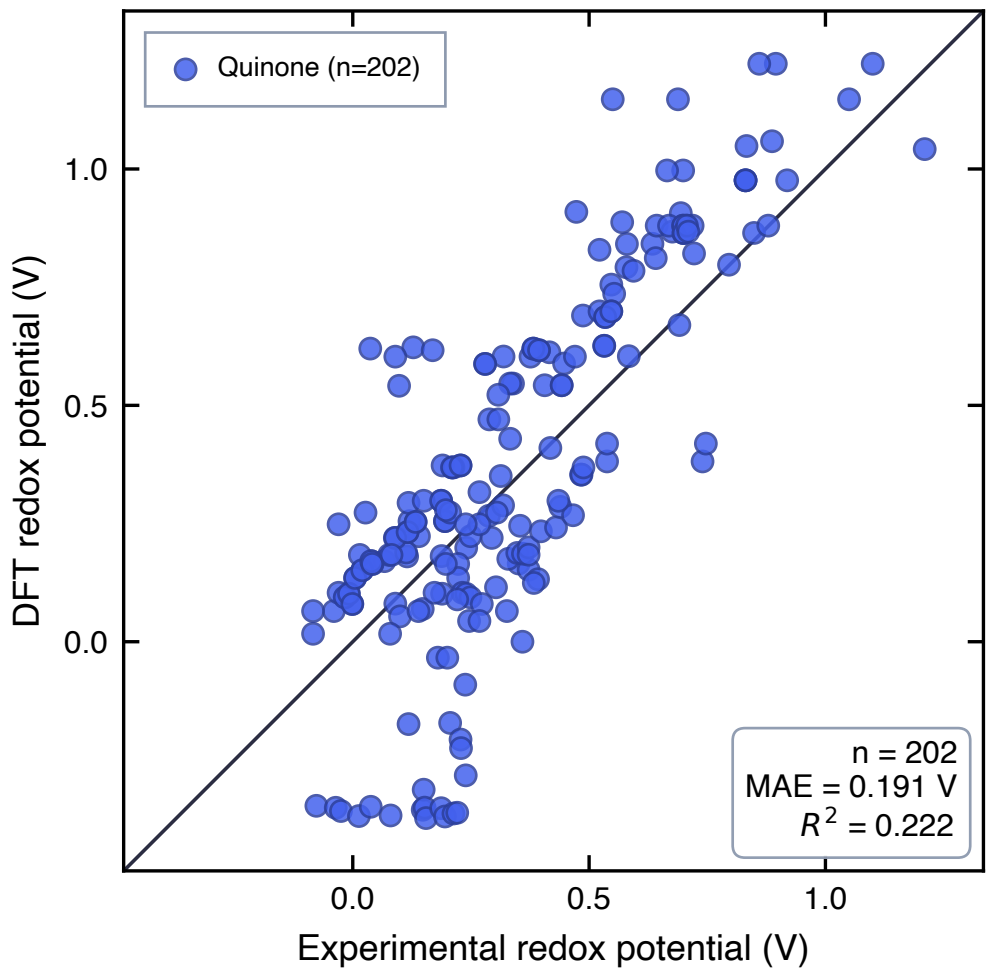

(a) Quinones

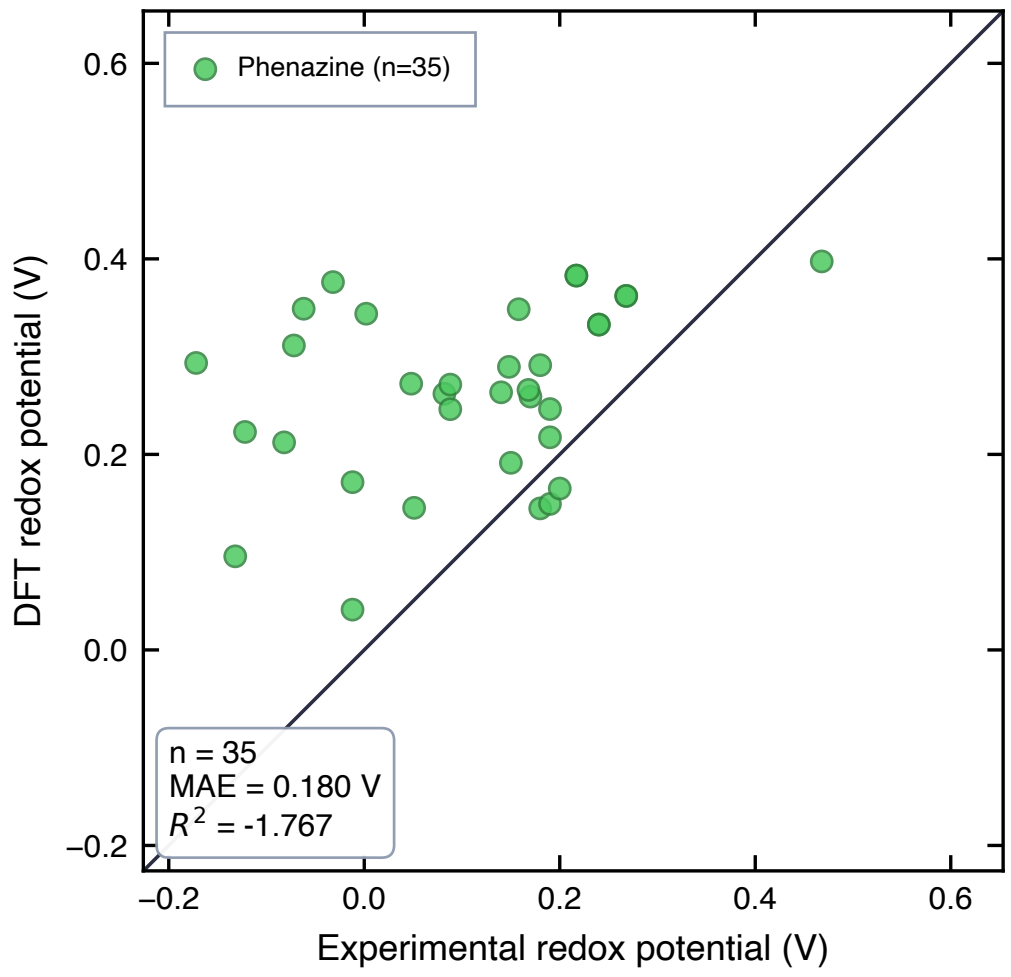

(b) Phenazines

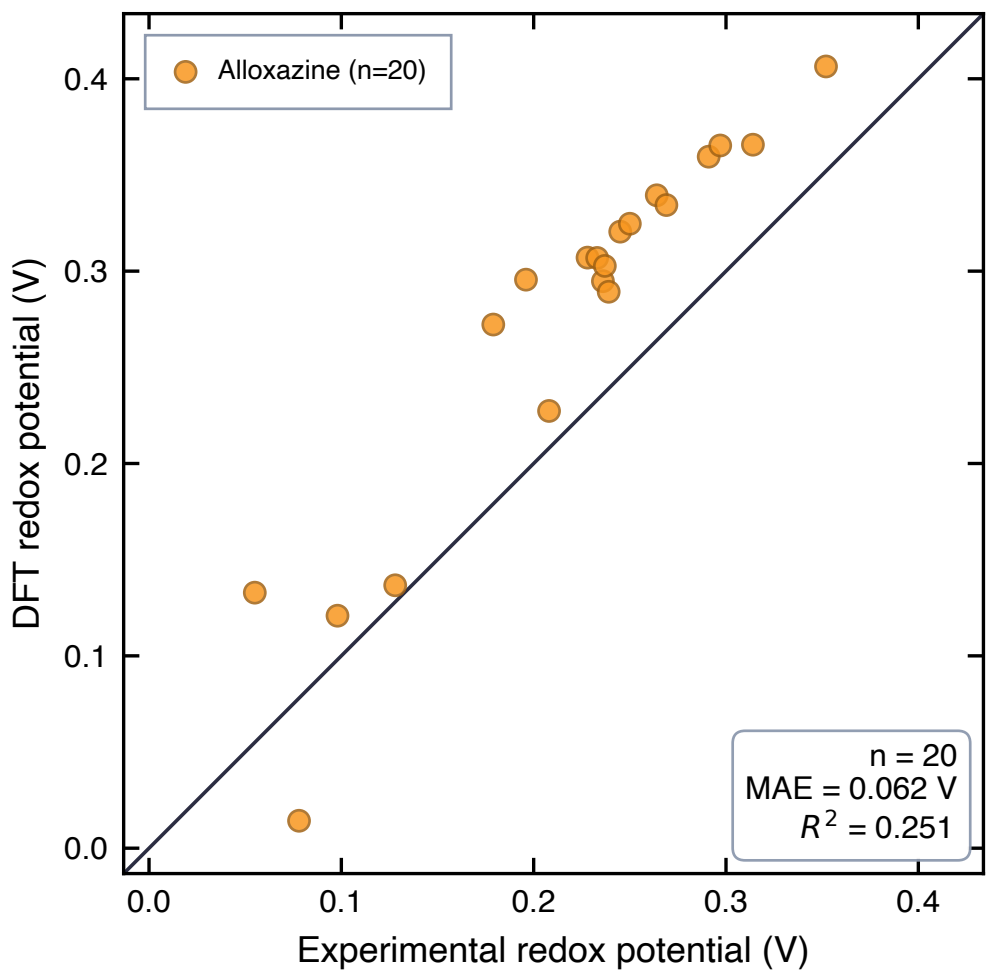

(c) Alloxazines

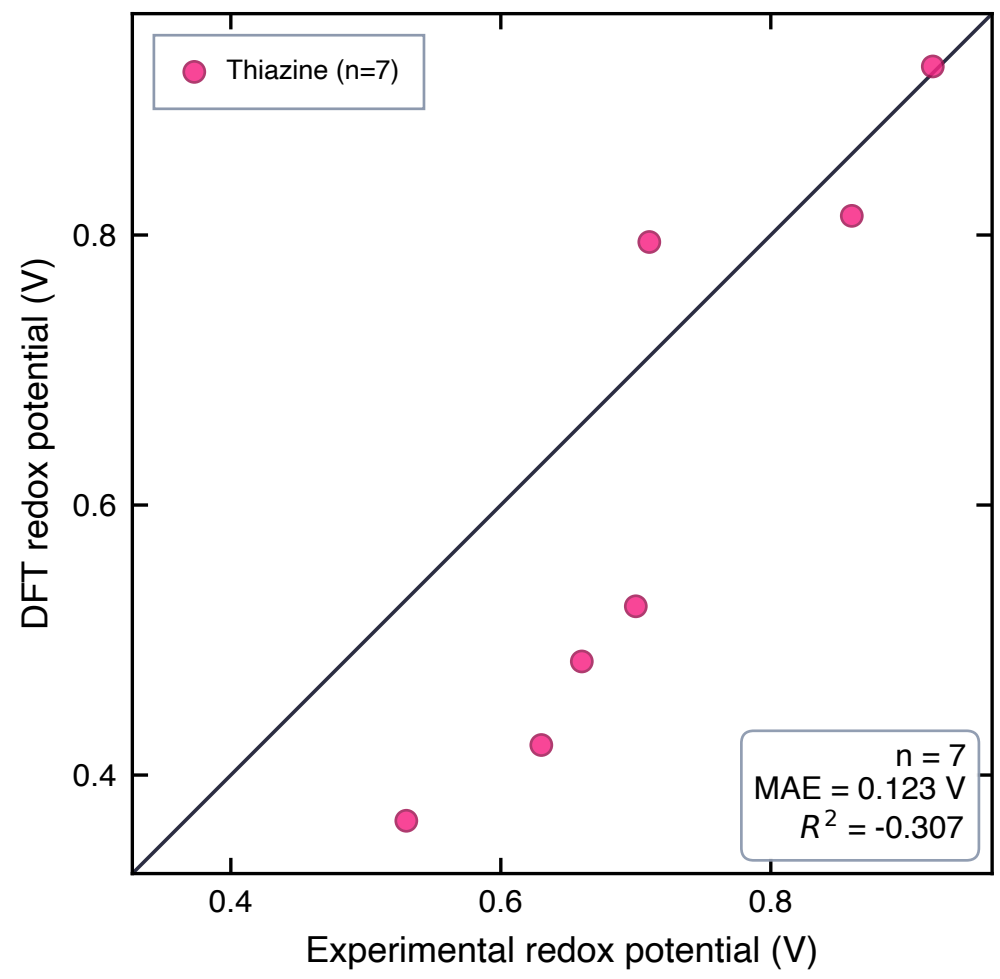

(d) Thiazines

Figure S6: Direct comparison between M06-2X DFT-calculated and experimentally measured redox potentials for the individual (a) quinone, (b) phenazine, (c) alloxazine and (d) thiazine classes. The diagonal line represents perfect agreement between calculated and experimental redox potentials.

To test whether the DFT–experimental discrepancy could be described by a systematic offset, a constant correction was calculated for each chemical class:

$$\Delta_{\mathrm{class}} = \mathrm{mean}\left(E^{\circ}_{\mathrm{exp}} - E^{\circ}_{\mathrm{DFT}}\right), \tag{1}$$

and applied according to:

$$E^{\circ}_{\mathrm{DFT,corr}} = E^{\circ}_{\mathrm{DFT}} + \Delta_{\mathrm{class}}. \tag{2}$$

This correction removes the mean signed bias within each class without changing the relative ordering of the DFT-calculated potentials.

Table S2: Effect of the class-specific offset correction on the agreement between DFT-calculated and experimental redox potentials. All 264 experimental measurements are included.

Class	n	Δ_{class} (V)	Raw MAE (V)	Corrected MAE (V)	Raw R^2	Corrected R^2
Quinone	202	-0.030	0.191	0.185	0.222	0.235
Phenazine	35	-0.170	0.180	0.117	-1.767	-0.227
Alloxazine	20	-0.056	0.062	0.025	0.251	0.785
Thiazine	7	+0.097	0.123	0.096	-0.307	0.303

The offset correction has different effects across the four chemical classes. For quinones, the offset is small ($\Delta_{class} = -0.030$ V), and the MAE changes only slightly from 0.191 to 0.185 V. For phenazines, applying an offset of -0.170 V reduces the MAE from 0.180 to 0.117 V. The largest improvement is observed for alloxazines, where a correction of -0.056 V reduces the MAE from 0.062 to 0.025 V. For thiazines, the MAE decreases from 0.123 to 0.096 V, although only seven measurements are available. These results show that a class-specific offset accounts for part of the DFT–experimental difference for phenazines and alloxazines, but has little effect for quinones.

S2. Graph neural network architecture

A molecule is naturally represented as a graph $\mathrm{G} = (V, E)$, where each node $v \in V$ corresponds to an atom and each edge $(u, v) \in E$ to a bond. The graph is described by a node feature matrix $\mathbf{X}_\mathcal{G}$ (one row per atom) and an adjacency matrix $\mathbf{A}_\mathcal{G}$, where $A_{uv} = 1$ if atoms u and v are bonded.[2] Each atom can be initialised with a feature vector $\mathbf{h}_v^{(0)} \in \mathbb{R}^{d_v}$ encoding chemical identity such as atomic number or hybridisation. Each bond carries a feature vector $\mathbf{e}_{uv} \in \mathbb{R}^{d_e}$ encoding bond-level attributes.

Graph neural networks (GNNs) learn atom-level representations through K rounds of *message passing*, learning information in each round, during which two operations are applied to every atom v:

Aggregate: atom v collects the current feature vectors of all its directly bonded neighbours $u \in \mathcal{N}(v)$, together with the corresponding bond features $\mathbf{e}_{uv}$, and combines them into a single message vector $\mathbf{m}_v^{(k)}$:

$$\mathbf{m}_v^{(k)} = \mathrm{AGG}^{(k)}\Big(\Big\{\, \mathbf{h}_u^{(k-1)}, \mathbf{e}_{uv} : u \in \mathcal{N}(v) \,\Big\}\Big) . \tag{3}$$

Importantly, the aggregation function AGG must be permutation-invariant as the neighbours have no natural ordering.[3]

Update: atom v uses the aggregated message $\mathbf{m}_v^{(k)}$ alongside its own current embedding $\mathbf{h}_v^{(k-1)}$ to produce a new embedding $\mathbf{h}_v^{(k)}$ usally via a multi-layer perceptron (MLP).

To predict a molecular property such as the redox potential, all atom embeddings are aggregated into a single molecular representation by a permutation-invariant readout R:

$$\mathbf{h}_\mathcal{G} = R\Big(\Big\{\, \mathbf{h}_v^{(K)} \;\Big|\; v \in \mathcal{V} \,\Big\}\Big) . \tag{4}$$

The resulting vector $\mathbf{h}_\mathcal{G} \in \mathbb{R}^{d_h}$ serves as a holistic molecular descriptor, which is passed to an MLP prediction head to produce the final prediction.

We evaluate five GNN architectures that differ purely in how they gather information from neighbours (**3**). Graph Convolutional Network GCN serves as our standard baseline, treating all neighbouring atoms equally during the update.[4] GATv2 is a more expressive version of Graph attention network (GAT), with stronger performance as highlighted by Brody et al.[5] GAT models assign attention to specific structural motifs, allowing it to give higher weights to chemically important atoms, which aligns with chemical intuition for redox potentials. Graph isomorphism network (GIN) is designed to distinguish between subtle, structural isomers that simpler architectures such as GCN might view as identical.[6] GraphSAGE separates the central atom's identity from its neighbours during the update, aiding model generalisation.[6] Directed message-passing neural network (D-MPNN) passes information along chemical bonds rather than atoms which is well-suited for capturing the electronic effects in redox molecules dictating redox potential.[7] All GNN models in this paper implement the same featurisation, readout function, MLP and are deployed with PyTorch Geometric.[8]

S3. Featurisation

Atom and bond features are extracted using RDKit. Tables S3 and S4 list the full feature sets.

Table S3: Node (atom) features. One-hot features include an additional "unknown" bin. Continuous features are standardised to zero mean and unit variance using training-set statistics.

Feature	Encoding	Detail	Dim
Atomic number	One-hot	28 elements (H, Li, B, C, N, O, F, Na, Mg, Al, Si, P, S, Cl, K, Ca, V, Cr, Mn, Fe, Co, Ni, Cu, Zn, Se, Br, Mo, I) + unknown	29
Degree	One-hot	0–5 + unknown	7
Hybridisation	One-hot	Unspecified, S, SP, SP2, SP3, SP2D, SP3D, SP3D2, Other + unknown	10
Chirality	One-hot	Unspecified, CW, CCW, Other, Tetrahedral, Allene, Squareplanar, Trigonalbipyramidal, Octahedral + unknown	10
Total H count	One-hot	0–4 + unknown	6
Aromaticity	Binary	1 if atom is aromatic, 0 otherwise	1
Ring membership	Binary	1 if atom is in any ring, 0 otherwise	1
Ring size	Binary	One bit per ring size 3, 4, 5, 6, 7, 8	6
Formal charge	Continuous	Raw integer charge	1
Atomic mass	Continuous	Atomic mass in Da	1
Total valence	Continuous	Total valence	1
Radical electrons	Continuous	Number of radical electrons	1
		**Total**	**74**

Table S4: Edge (bond) features. Edges are stored in both directions with each direction carrying the same feature vector.

Feature	Encoding	Detail	Dim
Bond type	Binary	One bit each for single, double, triple, aromatic	4
Conjugated	Binary	1 if bond is conjugated, 0 otherwise	1
In ring	Binary	1 if bond is in a ring, 0 otherwise	1
Stereo	One-hot	None, Any, Z, E, Cis, Trans, AtropCW, AtropCCW + unknown	9
		**Total**	**15**

S4. Pre-training

S4.1 Pre-training Hyperparameter Optimisation

Table S5: Pre-training hyperparameter search space and best values from 50-trial hyperparameter optimisation (HPO). All models have sigmoid-weighted readout, ReLU activation and seed = 42.

**Hyperparameter**	**Range**	**GCN**	**GATv2**	**GIN**	**GraphSAGE**	**D-MPNN**
Hidden dimension	$\{64, 128, 256, 512\}$	128	256	128	64	64
Number of layers	$[2, 5]$	4	5	4	4	3
Learning rate	$[10^{-4}, 10^{-2}]$	9.5×10^{-3}	5.7×10^{-4}	3.5×10^{-3}	4.8×10^{-3}	2.2×10^{-3}
Weight decay	$[10^{-6}, 10^{-3}]$	1.4×10^{-4}	1.7×10^{-4}	4.7×10^{-5}	1.2×10^{-5}	5.3×10^{-5}
Batch size	$\{32, 64, 128\}$	64	64	64	64	64
Dropout	$[0.0, 0.4]$	1.3×10^{-3}	2.5×10^{-2}	1.3×10^{-3}	1.4×10^{-3}	6.9×10^{-3}
Model-specific:						
Attention heads	$\{2, 4, 8\}$	—	2	—	—	—
Aggregation	{mean, max, add}	—	—	—	max	—

S5. Fine-tuning

S5.1 Fine-tuning HPO

The search space and best hyperparameters are shown in table **S6** (no pre-training), table **S7** (full fine-tuning), table **S8** (partial fine-tuning), and table **S9** (readout + head).

Table S6: No-pre-training baseline: HPO search space and best values. Models trained from random initialisation.

**Hyperparameter**	**Range**	**GCN**	**GATv2**	**GIN**	**GraphSAGE**	**D-MPNN**
Learning rate	$[10^{-5}, 10^{-2}]$	6.8×10^{-3}	1.8×10^{-3}	2.1×10^{-3}	3.6×10^{-3}	1.3×10^{-3}
Weight decay	$[10^{-6}, 10^{-3}]$	1.1×10^{-4}	1.2×10^{-4}	4.2×10^{-5}	4.9×10^{-5}	4.8×10^{-6}
Batch size	$\{16, 32, 64\}$	64	32	16	32	64
Dropout	$[0.0, 0.3]$	5.4×10^{-3}	3.7×10^{-2}	5.7×10^{-2}	4.7×10^{-2}	1.5×10^{-2}
**CV MAE (V)**		**0.091**	**0.087**	**0.098**	**0.087**	**0.105**

Table S7: Full fine-tuning: HPO search space and best values. Freeze ratio = 0 (all layers initialised from pre-trained checkpoint, all parameters updated).

**Hyperparameter**	**Range**	**GCN**	**GATv2**	**GIN**	**GraphSAGE**	**D-MPNN**
Learning rate	$[10^{-5}, 10^{-2}]$	7.1×10^{-3}	4.3×10^{-3}	4.5×10^{-3}	1.0×10^{-3}	2.0×10^{-3}
Weight decay	$[10^{-6}, 10^{-3}]$	5.2×10^{-5}	2.0×10^{-5}	9.9×10^{-5}	1.6×10^{-4}	2.9×10^{-6}
Batch size	$\{16, 32, 64\}$	64	64	64	64	16
Dropout	$[0.0, 0.3]$	1.5×10^{-2}	2.2×10^{-2}	2.9×10^{-2}	1.1×10^{-1}	1.6×10^{-2}
**CV MAE (V)**		**0.078**	**0.076**	**0.077**	**0.081**	**0.080**

Table S8: Partial fine-tuning: HPO search space and best values. Freeze ratio = 0.5 (bottom half of conv layers frozen).

**Hyperparameter**	**Range**	**GCN**	**GATv2**	**GIN**	**GraphSAGE**	**D-MPNN**
Learning rate	$[10^{-5}, 10^{-2}]$	9.1×10^{-3}	2.7×10^{-3}	8.8×10^{-3}	2.2×10^{-3}	1.8×10^{-3}
Weight decay	$[10^{-6}, 10^{-3}]$	6.5×10^{-6}	2.4×10^{-5}	7.6×10^{-5}	3.9×10^{-5}	2.9×10^{-4}
Batch size	$\{16, 32, 64\}$	64	64	64	64	64
Dropout	$[0.0, 0.3]$	2.3×10^{-2}	5.3×10^{-2}	2.6×10^{-3}	6.6×10^{-2}	2.1×10^{-2}
**CV MAE (V)**		**0.084**	**0.077**	**0.078**	**0.077**	**0.084**

Table S9: Readout + head fine-tuning: HPO search space and best values. Freeze ratio = 1.0 (all conv layers frozen, only readout and prediction head trained).

**Hyperparameter**	**Range**	**GCN**	**GATv2**	**GIN**	**GraphSAGE**	**D-MPNN**
Learning rate	$[10^{-5}, 10^{-2}]$	6.4×10^{-3}	6.3×10^{-3}	6.8×10^{-3}	3.2×10^{-3}	5.2×10^{-3}
Weight decay	$[10^{-6}, 10^{-3}]$	1.1×10^{-6}	1.8×10^{-6}	1.1×10^{-4}	2.2×10^{-4}	1.2×10^{-6}
Batch size	$\{16, 32, 64\}$	64	64	64	16	16
Dropout	$[0.0, 0.3]$	1.4×10^{-1}	5.1×10^{-2}	5.4×10^{-3}	4.4×10^{-2}	3.8×10^{-2}
**CV MAE (V)**		**0.101**	**0.083**	**0.086**	**0.089**	**0.085**

S5.2 Tabular baselines

Three tree-based models were trained as non-graph baselines: Random Forest, XGBoost, and CatBoost.[9,10] All used 2048-bit ECFP4 (Morgan) fingerprints generated with RDKit[11] as input features. Hyperparameters were optimised with Optuna[12] (50 trials, TPE sampler) using the same 5-fold grouped CV and train/test split as the GNN models. The search spaces and best hyperparameters are shown in table **S10**.

Table S10: Tabular baseline HPO search spaces and best values (bold). CV MAE from 5-fold grouped CV on the experimental data.

Model	Hyperparameter	Range	Best
Random Forest	n_estimators	$[100, 1000]$	**302**
	max_depth	$[3, 30]$	**24**
	min_samples_split	$[2, 20]$	**2**
	min_samples_leaf	$[1, 10]$	**1**
	max_features	{sqrt, log2, 0.3, 0.5, 0.8}	**0.8**
		**CV MAE (V)**	**0.116**
XGBoost	n_estimators	$[100, 1000]$	**284**
	max_depth	$[3, 12]$	**9**
	learning_rate	$[10^{-3}, 0.3]$	**0.022**
	subsample	$[0.5, 1.0]$	**0.60**
	colsample_bytree	$[0.3, 1.0]$	**0.62**
	reg_alpha	$[10^{-8}, 10]$	—
	reg_lambda	$[10^{-8}, 10]$	—
		**CV MAE (V)**	**0.114**
CatBoost	iterations	$[100, 1000]$	**576**
	depth	$[3, 10]$	**7**
	learning_rate	$[10^{-3}, 0.3]$	**0.093**
	l2_leaf_reg	$[10^{-3}, 10]$	**0.016**
	bagging_temperature	$[0, 1]$	**0.81**
		**CV MAE (V)**	**0.111**

S5.3 Experimental test set results

Table S11: Tabular baselines and GNN no-pretrain models on the experimental test set (n = 36 molecules). σ_{ens} = predictive uncertainty.

**Model**	**Type**	**MAE (V)**	R^2	σ_{ens}
RF (HPO)	Tabular	0.1036	0.627	0.0305
XGBoost (HPO)	Tabular	0.0850	0.730	0.0345
CatBoost (HPO)	Tabular	0.0913	0.735	0.0281
GCN	GNN	0.0697	0.831	0.0489
GATv2	GNN	0.0750	0.816	0.0423
GIN	GNN	0.0896	0.720	0.0695
GraphSAGE	GNN	0.0756	0.825	0.0642
D-MPNN	GNN	0.0947	0.663	0.0701

S5.4 Fine-tuning strategy ablation

Table S12: Fine-tuning strategy ablation (n = 36 molecules). MAE and R^2: mean ± std across the 5-fold ensemble. σ_{ens} = predictive uncertainty.

**Model**	**Strategy**	**MAE (V)**	R^2	σ_{ens}
GCN	No pre-training	0.0697 ± 0.0090	0.831 ± 0.048	0.0489
	Full fine-tuning	0.0619 ± 0.0081	0.874 ± 0.058	0.0432
	Partial fine-tuning	0.0644 ± 0.0034	0.868 ± 0.016	0.0359
	Readout + head	0.0795 ± 0.0043	0.776 ± 0.037	0.0325
GATv2	No pre-training	0.0750 ± 0.0070	0.816 ± 0.044	0.0423
	Full fine-tuning	0.0590 ± 0.0077	0.887 ± 0.026	0.0419
	Partial fine-tuning	0.0567 ± 0.0069	0.900 ± 0.025	0.0391
	Readout + head	0.0869 ± 0.0076	0.708 ± 0.064	0.0323
GIN	No pre-training	0.0896 ± 0.0125	0.720 ± 0.117	0.0695
	Full fine-tuning	0.0562 ± 0.0111	0.888 ± 0.077	0.0508
	Partial fine-tuning	0.0603 ± 0.0076	0.874 ± 0.050	0.0467
	Readout + head	0.0627 ± 0.0125	0.863 ± 0.072	0.0477
GraphSAGE	No pre-training	0.0756 ± 0.0186	0.825 ± 0.102	0.0642
	Full fine-tuning	0.0596 ± 0.0102	0.875 ± 0.082	0.0357
	Partial fine-tuning	0.0740 ± 0.0121	0.806 ± 0.068	0.0363
	Readout + head	0.0748 ± 0.0091	0.813 ± 0.044	0.0361
D-MPNN	No pre-training	0.0947 ± 0.0247	0.663 ± 0.226	0.0701
	Full fine-tuning	0.0757 ± 0.0092	0.790 ± 0.074	0.0625
	Partial fine-tuning	0.0671 ± 0.0097	0.832 ± 0.066	0.0469
	Readout + head	0.0604 ± 0.0079	0.857 ± 0.039	0.0440

References

[1] C. G. Cannon, P. A. A. Klusener, L. F. Petit, T. Wong, A. Wang, Q. Song, N. P. Brandon and A. R. J. Kucernak, *ACS Applied Energy Materials*, 2024.

[2] J. Zhou, Y. Yang, A. M. Mroz and K. E. Jelfs, *Digital Discovery*, 2025, **4**, 149–160.

[3] J. Zhou, G. Cui, S. Hu, Z. Zhang, C. Yang, Z. Liu, L. Wang, C. Li and M. Sun, *AI Open*, 2020, **1**, 57–81.

[4] S. Zhang, H. Tong, J. Xu and R. Maciejewski, *Computational Social Networks*, 2019, **6**, 11.

[5] S. Brody, U. Alon and E. Yahav, *How Attentive are Graph Attention Networks?*, 2022, `http://arxiv.org/abs/2105.14491`, arXiv:2105.14491 [cs].

[6] K. Xu, W. Hu, J. Leskovec and S. Jegelka, *How Powerful are Graph Neural Networks?*, 2019, `http://arxiv.org/abs/1810.00826`, arXiv:1810.00826 [cs].

[7] E. Heid, K. P. Greenman, Y. Chung, S.-C. Li, D. E. Graff, F. H. Vermeire, H. Wu, W. H. Green and C. J. McGill, *Journal of Chemical Information and Modeling*, 2024, **64**, 9–17.

[8] M. Fey and J. E. Lenssen, *Fast Graph Representation Learning with PyTorch Geometric*, 2019, `http://arxiv.org/abs/1903.02428`, arXiv:1903.02428 [cs].

[9] T. Chen and C. Guestrin, Proceedings of the 22nd ACM SIGKDD International Conference on Knowledge Discovery and Data Mining, 2016, pp. 785–794.

[10] F. Pedregosa, F. Pedregosa, G. Varoquaux, G. Varoquaux, N. Org, A. Gramfort, A. Gramfort, V. Michel, V. Michel, L. Fr, B. Thirion, B. Thirion, O. Grisel, O. Grisel, M. Blondel, P. Prettenhofer, P. Prettenhofer, R. Weiss, V. Dubourg, V. Dubourg, J. Vanderplas, A. Passos, A. Tp and D. Cournapeau, *MACHINE LEARNING IN PYTHON*.

[11] G. Landrum, *RDKit: Open-source cheminformatics*, `https://www.rdkit.org/`.

[12] T. Akiba, S. Sano, T. Yanase, T. Ohta and M. Koyama, *Optuna: A Next-generation Hyperparameter Optimization Framework*, 2019, `http://arxiv.org/abs/1907.10902`, arXiv:1907.10902 [cs].
